# Enrichment of CD4^−^CD8^−^ cytotoxic Vδ1/3 T cells in pulmonary tuberculosis disease

**DOI:** 10.1016/j.isci.2026.117241

**Published:** 2026-08-19

**Authors:** Kendall Kearns, Rashmi Tippalagama, Ashu Chawla, Jason Greenbaum, Aruna Dharshan De Silva, Wathsala Gunasinghe, Judy Perera, Hansani Gunasekera, Darsha D. Senevirathne, Thomas J. Scriba, Julie G. Burel, Cecilia S. Lindestam Arlehamn, Bjoern Peters

**Affiliations:** 1 La Jolla Institute for Immunology, La Jolla, CA 92037, USA; 2 University of California, San Diego, La Jolla, CA 92037, USA; 3 General Sir John Kotelawala Defence University, Colombo 10390, Sri Lanka; 4 National Hospital for Respiratory Diseases, Welisara 11010, Sri Lanka; 5 South African Tuberculosis Vaccine Initiative, University of Cape Town, Cape Town 7935, South Africa; 6 Center for Vaccine Research, Department of Infectious Disease Immunology, Statens Serum Institut, 2300 Copenhagen, Denmark

**Keywords:** tuberculosis, γδ T cells, single-cell RNA sequencing, TB treatment

## Abstract

Tuberculosis (TB), caused by *Mycobacterium tuberculosis* (*Mtb*), remains a leading cause of global morbidity and mortality. Although gamma-delta (γδ) T cells have increasingly been suggested to contribute to the TB immune response, quantitative and qualitative differences in this immune cell compartment between healthy and diseased individuals are not well-characterized. In this study, we used single-cell RNA sequencing to provide a high-resolution characterization of CD4^−^CD8^−^ γδ T cells in peripheral blood across healthy *Mtb*-(non-)sensitized and TB disease pre-/post-treatment cohorts. We found an activated and cytotoxic gene signature in γδ T cells of TB disease compared with both healthy cohorts. Strikingly, these differences persisted through 1 year following TB disease diagnosis. These transcriptomic differences were largely mediated by an NK-like cytotoxic Vδ1 and Vδ3 subset that was enriched in TB disease. Our findings suggest long-lasting changes in the CD4^−^CD8^−^ γδ T cell compartment and highlight specific subsets as potentially important in TB.

## Highlights

• CD4^−^CD8^−^ γδ T cells exhibit persistent activated profile after TB treatment
• NK-like cytotoxic Vδ1/3 cells are enriched in tuberculosis disease
• *TRGV4* TCR usage is increased in TB-associated Vδ1/3 cells
• TB-associated γδ T cell changes persist 1 year after diagnosis

## Graphical abstract

**Figure:**
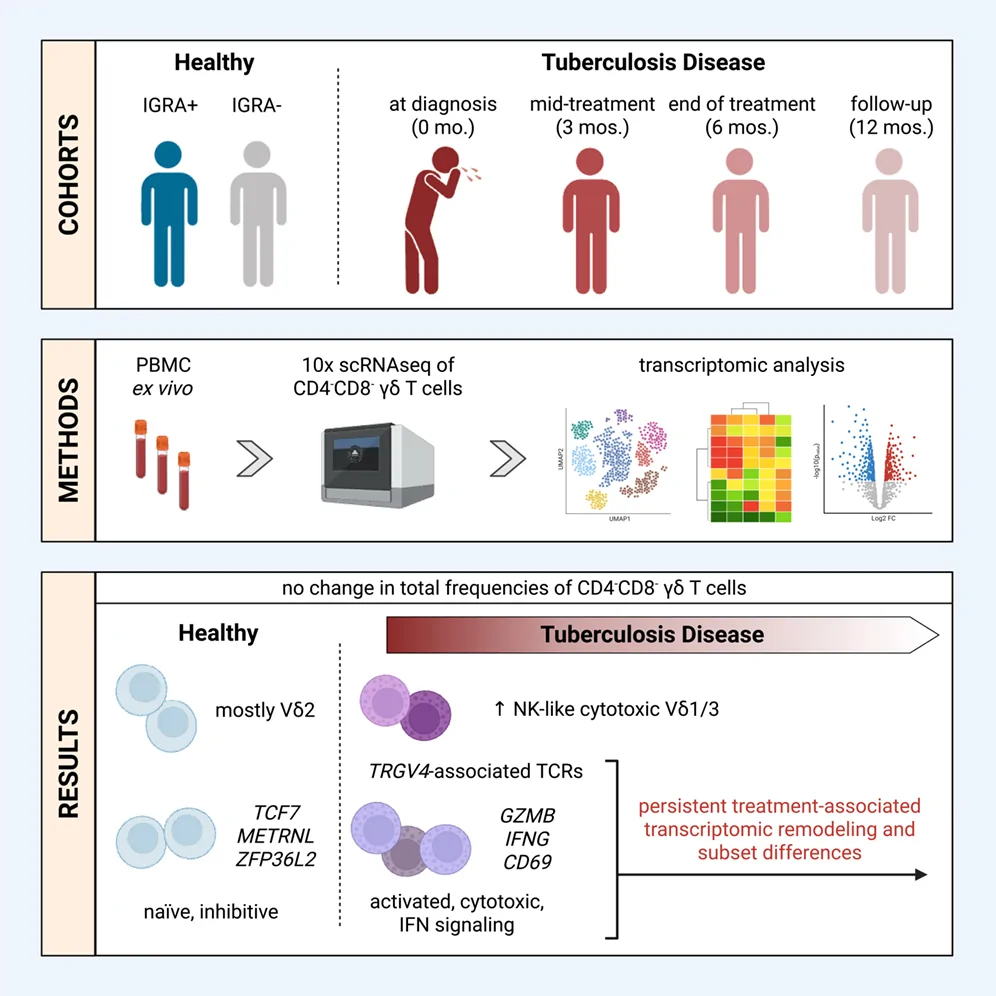

## Teaser

Immunology; Omics

## Introduction

Tuberculosis (TB), an airborne infection caused by *Mycobacterium tuberculosis* (*Mtb*), is reported to affect over 10 million people annually and remains a leading cause of death worldwide, despite the availability of antibiotic treatments and the Bacillus Calmette-Guérin (BCG) vaccine.^1^ Conventional alpha-beta (αβ) CD4 T cells recognizing peptide antigens are well established as the primary lymphocyte subset involved in protective immunity to TB.^2^ However, growing evidence supports a role for unconventional T cells in the TB immune response. In our previous studies aimed at identifying peptide epitopes in *Mtb*,^3^^,^^4^ we noted IFNγ production in CD4^−^CD8^−^ T cells stimulated with *Mtb* extracts that was not present after *Mtb*-derived peptide stimulation. Here, we wanted to characterize the CD4^−^CD8^−^ T cell population, of which γδ T cells comprise a major proportion, in response to stimulation with *Mtb* antigens and *ex vivo* at the transcriptomic level across TB cohorts.

γδ T cells differ from conventional αβ T cells in that their T cell receptors (TCR) are encoded by a different set of genes and they are largely CD4^−^CD8^−^ instead of either CD4^+^ or CD8^+^.^5^ They have been reported to be able to respond to non-peptide antigens and may not be MHC restricted.^6^^,^^7^ They also hold features of both innate and adaptive immune cells, combining rapid responses with specificity, poising γδ T cells as potentially important in early stages of *Mtb* infection. The two predominant γδ T cell subsets are Vδ1 and Vδ2, which are named by their expression of *TRDV1* and *TRDV2* TCR genes, respectively, though other smaller subsets like Vδ3 (expressing *TRDV3*) exist.^8^ Vδ2 cells are the most prominent in circulation, of which >90% have Vγ9Vδ2 TCR pairing.^9^ Vγ9Vδ2 cells are known to recognize microbe-derived phosphoantigens, like HMBPP, that are present in *Mtb* and BCG.^10^ Vδ1 cells are less common in the periphery but are found at higher frequencies in tissues such as the mucosa.^9^ Vδ1 cells have been found to be enriched in the lungs of individuals with TB disease, suggesting they may be poised to quickly respond to *Mtb* challenge.^11^ Similarly, Vδ3 cells are rarely detected in circulation but can be found at higher frequencies in tissues like the liver and gut.^8^

As Vδ2 cells are the most prominent γδ T cell subset in the circulation, they are the most well-studied in the context of human TB infection. Interestingly, several studies have demonstrated a significant decrease in the Vδ2:Vδ1 ratio in the circulation of individuals with TB disease compared with healthy individuals without evidence for TB infection^11^^,^^12^ as well as in the lungs compared with individuals with sarcoidosis,^13^ suggesting potential induction of activation-induced cell death of Vδ2 T cells or expansion/migration of Vδ1 T cells during TB infection. In terms of functionality, γδ T cells can expand and produce IFNγ following BCG vaccination, provide memory-like responses, and protect against TB in nonhuman primate models, but may not be sufficient to clear the disease.^14^^,^^15^^,^^16^^,^^17^^,^^18^^,^^19^ γδ T cells also exhibit cytotoxic and regulatory functions, which may be involved in protection against TB.^15^^,^^20^ As these functions are critical in responding to TB infection, deeper characterization of γδ T cells would be beneficial in better understanding their roles in the TB immune response across disease states and anti-TB treatment.

Currently, there is an overall lack of comprehensive data on how γδ T cells and their phenotypes differ between TB cohorts and how they are affected by TB treatment. Several studies have analyzed γδ T cells with single-cell RNA sequencing (scRNAseq) in the context of TB, highlighting trained immunity and subset heterogeneity of γδ T cells.^15^^,^^21^^,^^22^ However, these studies were limited in cell numbers (<7,000 cells per study) and did not examine transcriptomic changes after starting anti-TB therapy. To build on this and further investigate the reactivity of CD4^−^CD8^−^ T cells across TB cohorts, we utilized blood samples from two healthy cohorts with or without *Mtb* sensitization and one TB disease cohort with longitudinal sampling at four timepoints pre- and post-treatment. We quantified IFNγ production by CD4^−^CD8^−^ T cells in response to different *Mtb* antigens and identified γδ T cells as the major source of IFNγ. We then isolated over 19,000 γδ T cells for scRNAseq analysis to further probe their transcriptomic profiles across TB cohorts. We hypothesized that this comprehensive dataset could help identify specific subsets of γδ T cells associated with TB disease compared with healthy cohorts with and without TB exposure as well as transcriptomic changes throughout anti-TB treatment.

## Results

### CD4^−^CD8^−^ γδ T cells respond to BCG but not an *Mtb*-derived peptide pool

**Figure 1:**
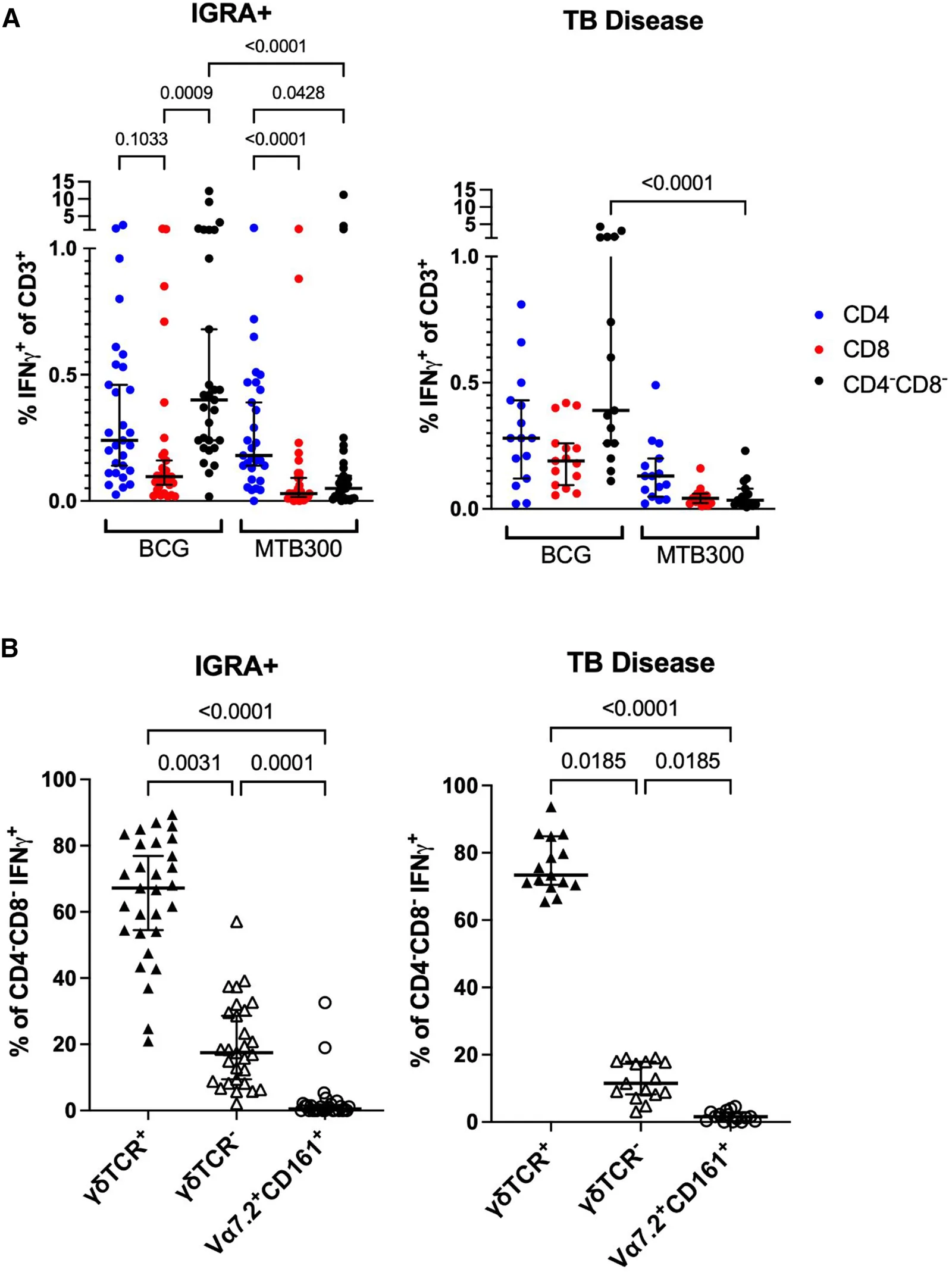
CD4^−^CD8^−^ γδ T cells produce IFNγ in response to BCG stimulation (A) Percent of IFNγ^+^ cells within the CD3^+^ population after stimulation with either BCG or MTB300 peptide pool separated by CD4^+^ (blue), CD8^+^ (red), and CD4^−^CD8^−^ (black) subsets. Samples isolated from IGRA+ and TB diseased individuals were utilized. Data are represented as median with 95% confidence interval. Friedman test with Dunn’s multiple comparisons test. (B) Percent of CD4^−^CD8^−^IFNγ^+^ cells stimulated with BCG that were γδTCR^+^, γδTCR^−^, or Vɑ7.2^+^CD161^+^ (MAIT cells) within the IGRA+ and TB disease cohorts. Data are represented as median with 95% confidence interval. Friedman test with Dunn’s multiple comparisons test. See also Figure S1.

To investigate reactivity to *Mtb* antigens across T cell populations, we compared IFNγ production in CD4^+^, CD8^+^, and CD4^−^CD8^−^ T cell subsets in response to *M. bovis* BCG and *Mtb*-derived peptide epitope pool (MTB300) stimulation.^3^ We isolated peripheral blood mononuclear cells (PBMCs) from individuals who were healthy *Mtb*-sensitized (determined by a positivity to IFNγ release assay, IGRA+) or those who had TB disease (active TB, ATB). PBMC samples were stimulated with either BCG or MTB300 and IFNγ production was measured after 12 h (Figure S1A). In the IGRA+ cohort, CD4^+^ T cells produced significantly more IFNγ than CD8^+^ T cells in response to both BCG and MTB300 stimulation (Figure 1A), supporting previous findings.^4^^,^^23^ We also found that CD4^−^CD8^−^ T cells produced more IFNγ than CD8^+^ T cells when stimulated with BCG but not MTB300 (Figure 1A, left), which was also observed in the TB disease cohort (Figure 1A, right). Characterization of the CD4^−^CD8^−^IFNγ^+^ population of both IGRA+ and ATB cohorts via flow cytometry revealed that it was predominantly γδ T cells and not MAIT cells (Vα7.2^+^CD161^+^) or other γδTCR^−^ subsets (Figure 1B). Similarly, IGRA− samples also exhibited IFNγ production by CD4^−^CD8^−^ T cells that were mostly γδ T cells (Figure S1B). This is in line with previous reports indicating that γδ T cells produce IFNγ in response to *Mtb* stimulation.^14^^,^^16^ These findings prompted further investigation of CD4^−^CD8^−^ γδ T cells in the context of TB.

### CD4^−^CD8^−^ γδ T cell frequencies do not change across TB cohorts and timepoints

**Figure 2:**
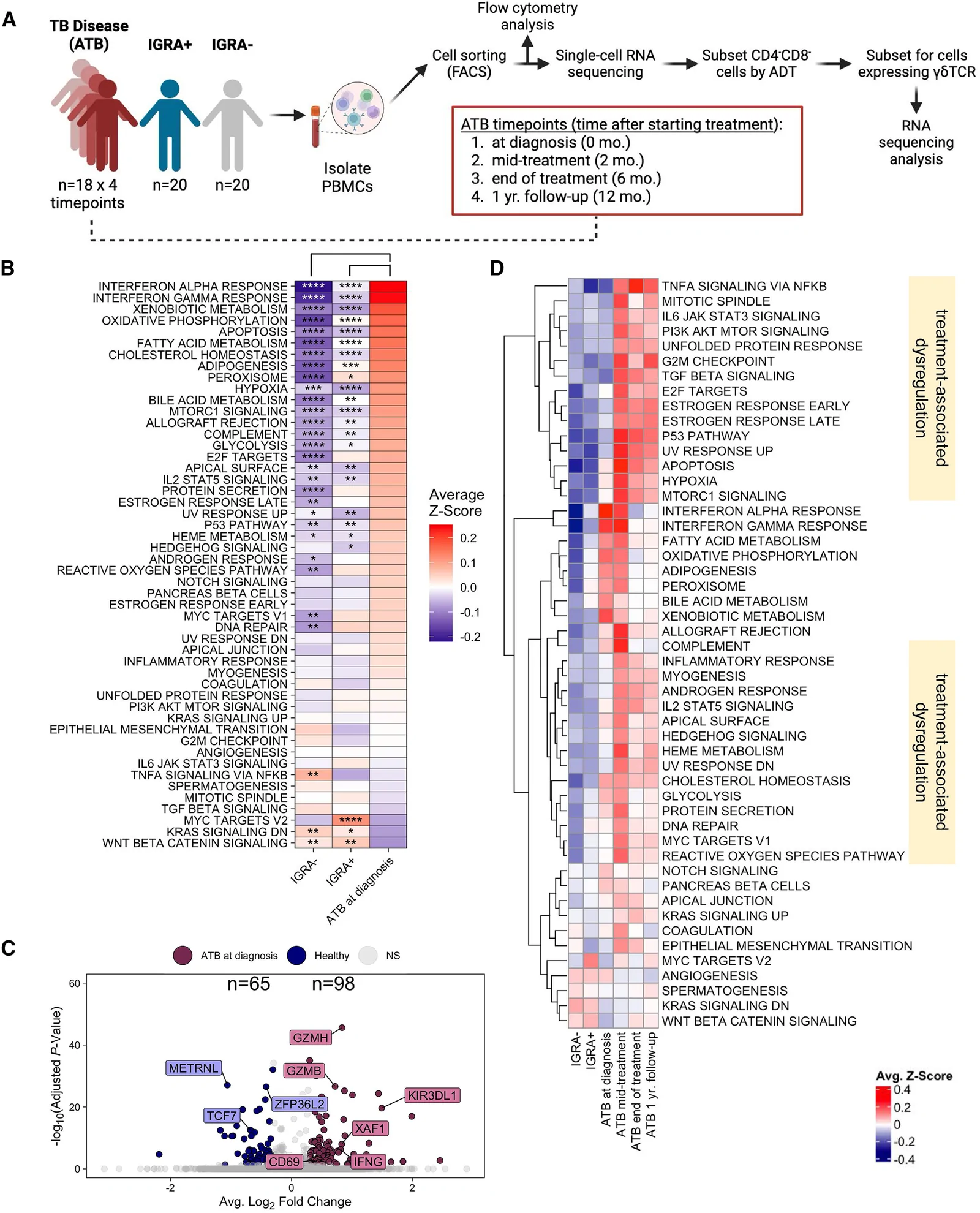
CD4^−^CD8^−^ γδ T cells from ATB and healthy cohorts are transcriptionally distinct (A) Experimental outline for examining CD4^−^CD8^−^ γδ T cells. Figure created with BioRender. (B) Enrichment of hallmark gene sets within the IGRA−, IGRA+, and ATB at diagnosis cohorts. Enrichment scores were calculated for each cell, scaled for each gene set to obtain *Z* scores, and the average *Z* score (plotted) was calculated per pathway within each cohort. The pathways are ordered by the difference in ATB at diagnosis average *Z* score and the average *Z* score of IGRA− and IGRA+ cohorts. Statistical tests were performed using pairwise Wilcoxon rank-sum tests with Benjamini-Hochberg multiple testing correction of *Z* scores between ATB at diagnosis and IGRA− or ATB at diagnosis and IGRA+ cohorts, with the significant adjusted *p* values displayed in the IGRA− and IGRA+ columns, respectively. ∗*p* < 0.05; ∗∗*p* < 0.01; ∗∗∗*p* < 0.001; ∗∗∗∗*p* < 0.0001. (C) Differential gene expression analysis between ATB at diagnosis (red) vs. IGRA−/IGRA+ (healthy, blue). Labeled genes include the gene with the highest fold change and genes selected based on biological relevance. (D) Average GSEA *Z* scores for all cohorts/timepoints. See also Tables S1, S2, and S3, as well as Figures S2, S3, S4, and S5.

To further investigate CD4^−^CD8^−^ γδ T cells, we measured their frequency using flow cytometry in PBMCs isolated from IGRA+ individuals and individuals with ATB across four timepoints throughout anti-TB treatment in addition to healthy *Mtb*-non-sensitized controls (IGRA−) (Figure 2A). We observed no significant differences in the frequencies of CD4^−^CD8^−^ γδ T cells between the cohorts and treatment timepoints (Figure S2A), biological sex (Figure S2B), and geographical locations (Figure S2C). Additionally, there were no significant differences in CD4^+^ and CD8^+^ γδ T cell population frequencies (Figure S2D). We also noted high variability across individuals within each cohort, which may be indicative of participant-intrinsic factors that influence γδ T cell frequencies.^24^^,^^25^ Thus, there were no differences in the frequency of total CD4^−^CD8^−^ γδ T cells across cohorts.

### CD4^−^CD8^−^ γδ T cells from ATB and healthy cohorts are transcriptionally distinct

To identify differences in transcriptional phenotypes between CD4^−^CD8^−^ γδ T cells isolated from individuals across ATB and healthy cohorts, we performed single-cell RNA sequencing (scRNAseq) on *ex vivo* fluorescence-activated sorted CD3^+^CD14^−^CD19^−^CD8^−^ T cells (Figures 2A and S3). Following scRNAseq, we filtered for cells that were CD4^−^ by antibody-derived tag (ADT), passed quality control metrics, and expressed γδTCR genes (STAR Methods; Figure S4), which resulted in 19,807 CD4^−^CD8^−^ γδ T cells used for downstream analyses.

First, we downsampled per sample due to variable cell numbers (Table S1) to perform single-cell gene set enrichment analysis (GSEA) with hallmark gene sets using the escape (v2.0.0) R package^26^ to identify global differences in CD4^−^CD8^−^ γδ T cells between the IGRA−, IGRA+, and ATB at diagnosis cohorts. We first focused on the ATB at diagnosis cohort, as it is the most acute timepoint and was expected to have the most differences in comparison with the healthy cohorts. Indeed, the pathway analysis showed fewer differences between healthy cohorts than to ATB at diagnosis (Figure S5A), suggesting that the IGRA− and IGRA+ cohorts are transcriptionally similar. Several pathways were statistically different between the IGRA− and IGRA+ cohorts, including lower expression of genes related to interferon alpha/gamma response and metabolism in IGRA− individuals, suggesting changes in transcriptomic profiles induced by prior exposure to *Mtb* (Figure S5A). Cells isolated from PBMC samples of the ATB at diagnosis cohort were significantly enriched for several immune activation pathways, including interferon alpha/gamma response and several metabolic pathways, compared with both IGRA− and IGRA+ cohorts (Figure 2B). Furthermore, we observed substantial overlap in leading-edge genes across cohorts for the interferon gamma response pathway (Table S2; Figure S5B). In cluster 0, 56 leading-edge genes were shared among IGRA−, IGRA+, and ATB at diagnosis, representing approximately 64% of the ATB leading-edge gene set. Additional genes were shared between ATB and either IGRA− or IGRA+, whereas a subset of genes remained cohort-specific (Figure S5B). These findings suggest that interferon gamma response represents a conserved transcriptional program within this cell population, with cohort-specific differences arising from variation in the relative contribution of individual genes rather than the emergence of an entirely distinct disease-associated pathway.

Since GSEA showed that the hallmark pathways were more similar between healthy cohorts than to ATB at diagnosis (Figures 2B and S5A), we performed differential gene expression analysis with the downsampled data to directly compare the two cohorts. This revealed a total of only nine differentially expressed genes between the IGRA− and IGRA+ cohorts (Figure S5C), further suggesting that these two groups were very similar based on transcriptional profiles. Thus, we combined the IGRA− and IGRA+ cohorts into a single “healthy” cohort for subsequent analysis. Using this combined healthy cohort, we performed a differential gene expression analysis with the ATB cohort. There were 65 and 98 significantly upregulated genes (DEGs) in the combined healthy cohort and ATB at diagnosis cohort, respectively (Figure 2C; Table S3). Healthy cohort upregulated DEGs included *TCF7*, *ZFP36L2*, and *METRNL*, which are associated with a naive and inhibitive phenotype. In the ATB at diagnosis cohort, DEGs were associated with an activated/cytotoxic phenotype (*IFNG*, *GZMB*, and *CD69*). These data suggest that TB disease significantly affects the transcriptomic profiles of circulating CD4^−^CD8^−^ γδ T cells, while those from healthy *Mtb*-sensitized and non-sensitized individuals are similar to each other.

### Long-lasting transcriptomic changes are observed post-*anti*-TB treatment

Next, we sought to determine whether the transcriptomic profiles of ATB samples collected after anti-TB treatment (mid-treatment [∼2 months], end of treatment [∼6 months], and post-treatment/1 year follow-up [∼12 months]) would gradually return to those of healthy samples. Indeed, for the interferon alpha/gamma response pathways again enriched in ATB at diagnosis (Figure 2D), we found a lower enrichment mid-treatment, which continued to decrease through the 1-year follow-up of ATB, suggesting that treatment reduced the amount of interferon alpha/gamma that CD4^−^CD8^−^ γδ T cells respond to. However, other pathways showed a treatment-associated dysregulation in samples collected after beginning anti-TB therapy compared with healthy cohorts and ATB at diagnosis, including apoptosis, reactive oxygen species pathway, and unfolded protein response (Figure 2D). 94 genes were upregulated in ATB 1 year follow-up compared with healthy samples, some of which were also upregulated in ATB at diagnosis (*IFNG* and *GZMB*), suggesting the activated transcriptomic profile persists 6 months after completing treatment (Figure S5D). Altogether, these data suggest that active *Mtb* infection and/or anti-TB therapy induce significant, long-lasting transcriptomic changes aligned with an activated/cytotoxic phenotype compared with healthy cohorts.

### Distinct γδ T cell clusters identified with scRNAseq

**Figure 3:**
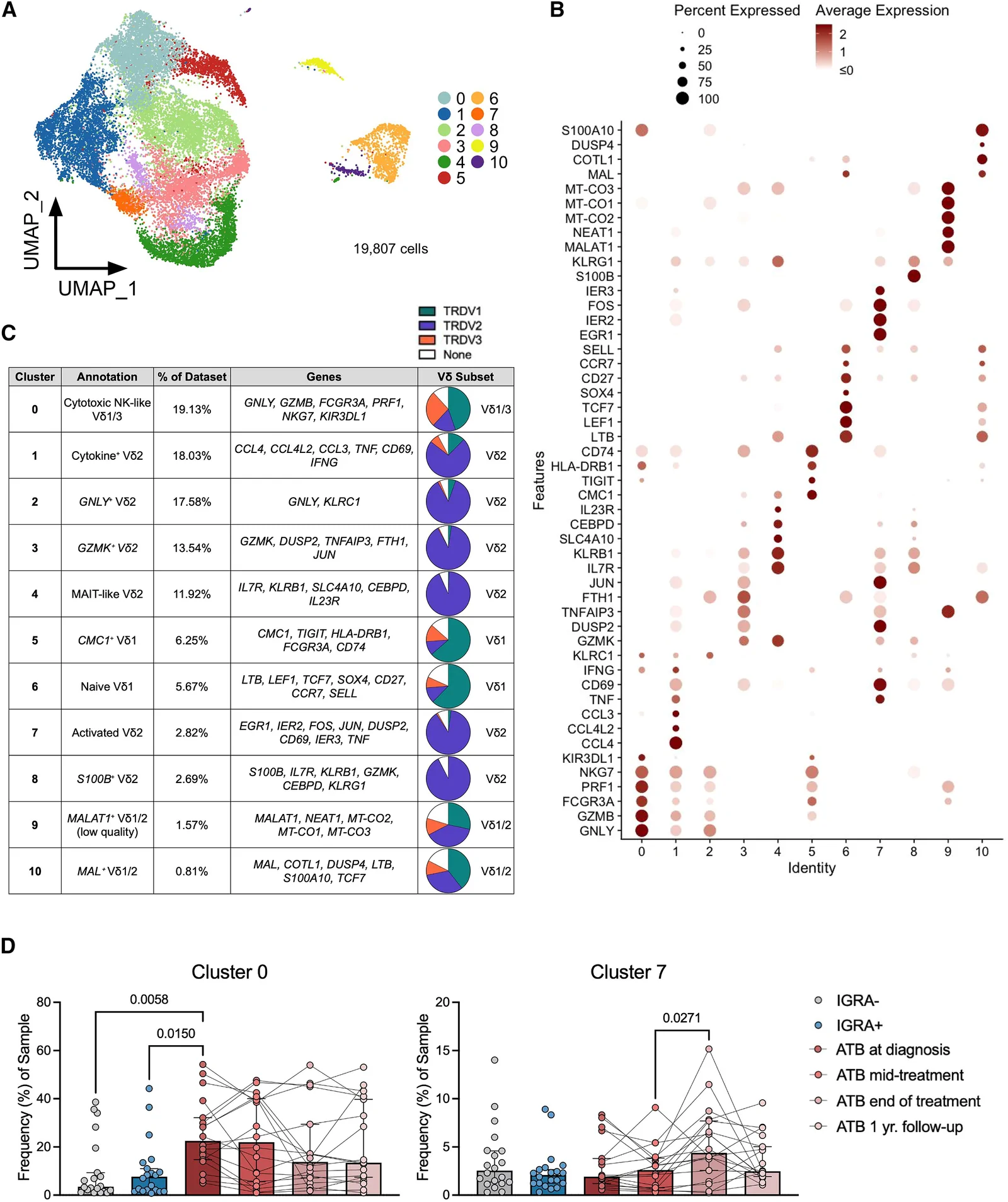
scRNAseq revealed distinct clusters of CD4^−^CD8^−^ γδ T cells (A) UMAP visualization of scRNA sequencing data colored by cluster. (B) Top expressed genes of each cluster (*y*) with the average expression and percent of cells expressing each gene per cluster (*x*). (C) Top genes, annotation, percent of dataset, Vδ subset, and proportion of cluster expressing *TRDV1*, *TRDV2*, or *TRDV3* (pie chart) for each cluster. A cell was determined to be *TRDV1*-, *TRDV2*-, or *TRDV3*-expressing if it had positive expression for one of the three genes and expression of 0 for the other two. A cluster was determined to be Vδ1, Vδ2, and/or Vδ3 if the associated gene was present in at least 25% of the cells within that cluster. (D) Frequency of each sample found in clusters 0 and 7. Data are represented as median with 95% confidence interval. For unpaired analyses, Kruskal-Wallis test with Dunn’s multiple comparisons test. For paired analyses, Friedman test with Dunn’s multiple comparisons test. See also Figure S6 and Tables S4 and S5.

To investigate heterogeneity within CD4^−^CD8^−^ γδ T cells, we performed unbiased clustering based on gene expression, which revealed 10 clusters (Figure 3A). The top-expressed genes were largely unique to each cluster (Figure 3B; Table S4). To annotate each cluster, we chose genes based on the gene with the highest average log_2_ fold change and other genes that had biological relevance per cluster as informed by Enrichr^27^^,^^28^^,^^29^ and manual identification (Figures 3C and S6A). Cluster 9 (*MALAT1*^+^ Vδ1/2) was determined to contain cells with low-quality transcriptomes based on higher expression of mitochondrial genes and lower numbers of features and RNA counts (Figure S6B) and was removed from downstream analyses.

We assigned a Vδ subset category to each cluster based on the proportion of cells expressing *TRDV1*, *TRDV2*, or *TRDV3* in each cluster (Figure 3C). The clusters were predominantly composed of either Vδ1 or Vδ2 cells, supporting previous findings that suggest the γδ T cell subsets are distinct populations.^30^^,^^31^ Additionally, most clusters were comprised of Vδ2 cells, which was expected as Vδ2 cells are the most prominent γδ T cell subset in circulation.^9^ Vδ2 clusters included cytokine^+^ Vδ2 (cluster 1), *GNLY*^+^ Vδ2 (cluster 2), *GZMK*^+^ Vδ2 (cluster 3), MAIT-like Vδ2 (cluster 4), activated Vδ2 (cluster 7), and *S100B*^+^ Vδ (cluster 8). Vδ1 clusters included *CMC1*^+^ Vδ1 (cluster 5) and naive Vδ1 (cluster 5). Several mixed clusters were also identified: cytotoxic NK-like Vδ1/3 (cluster 0), *MALAT1*^+^ Vδ1/2 (cluster 9), and *MAL*^+^ Vδ1/2 (cluster 10).

Next, we calculated the cell cluster composition in each sample and compared the results between cohorts (Figures 3D, S6C, and S6D). Broadly, there were few significant differences, including across ATB timepoints, though we noted increased frequencies of cluster 3 in IGRA− compared with IGRA+ individuals and the reverse for cluster 8 (Figure S6C). Cluster 7 showed a significant increase from ATB mid-treatment to end of treatment (Figure 3D), indicating treatment-related differences. Most clusters contained few differences between geographical locations, with the exception of cluster 4, which showed increased frequencies across all cohorts in South Africa compared with Sri Lanka (Figure S6D). Cluster 0 was the only cluster that showed significant enrichment in ATB compared with both IGRA− and IGRA+ healthy cohorts, which was corroborated through centered log ratio normalization (Figure 3D; Table S5). There were no significant longitudinal changes in cluster 0, though there was a trend for decreased frequencies through the 1 year follow-up timepoint. Overall, these data suggest potential involvement of cluster 0 (cytotoxic NK-like Vδ1/3) and cluster 7 (activated Vδ2) cell subsets in TB disease and resolution.

### Several clusters exhibit high ATB module scores and *IFNG* expression

**Figure 4:**
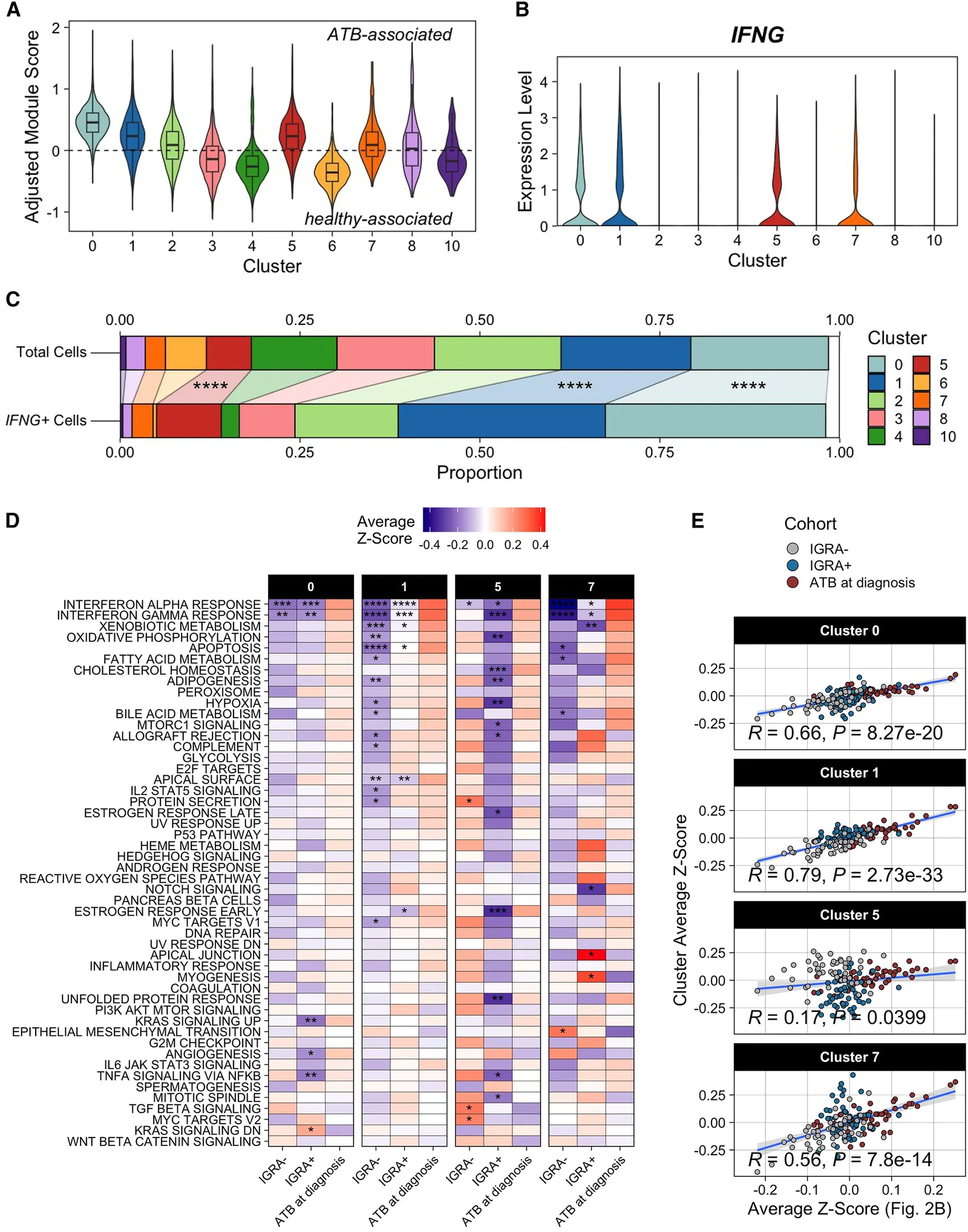
Several clusters exhibit high ATB module scores and IFNG expression (A) Module scores were calculated using the ATB- and healthy-associated genes in Figure 2C. The adjusted score was calculated by dividing the ATB score +1 by the healthy score +1, then taking the log2 of the result. Cluster 9 was removed due to low quality. (B) *IFNG* expression per cluster. Cluster 9 was removed due to low quality. (C) Proportion of the total cell population (left) or of *IFNG*+ cells (right) that originate from each cluster. Cells with an *IFNG* expression level of greater than 1 were classified as IFNG+. Exact binomial test of whether the proportion of each cluster within the *IFNG*+ cell subset is significantly greater than the proportion of each cluster within the total cell population. ∗*p* < 0.05; ∗∗*p* < 0.01; ∗∗∗*p* < 0.001; ∗∗∗∗*p* < 0.0001. (D) Enrichment of hallmark pathways within the IGRA−, IGRA+, and ATB at diagnosis cohorts for clusters 0, 1, 5, and 7. Pathways along the *y* axis are in the same order as in Figure 2B. Enrichment scores were calculated for each cell and were scaled for each gene set to obtain *Z* scores, which were then averaged within each cohort for each cluster. Statistical tests were performed using pairwise Wilcoxon rank-sum tests with Benjamini-Hochberg multiple testing correction of *Z* scores between ATB at diagnosis and IGRA− or ATB at diagnosis and IGRA+ cohorts, with the significant adjusted *p* values displayed in the IGRA− and IGRA+ columns, respectively. ∗*p* < 0.05; ∗∗*p* < 0.01; ∗∗∗*p* < 0.001; ∗∗∗∗*p* < 0.0001. (E) Plots showing the Pearson correlation between the average *Z* scores from Figure 2B (overall dataset) and from each cluster from the hallmark pathway enrichment analysis. See also Figure S7.

We next used the healthy vs. ATB at diagnosis DEGs (as reported in Figure 2C; Table S3) to determine whether any clusters had an ATB-associated signature. We calculated ATB and healthy module scores for each cell using the Seurat (v4.4.0) R package,^32^ then obtained the log_2_-transformed ratio to represent the relative enrichment of ATB- and healthy-associated DEGs. We calculated adjusted module scores for each cluster (Figure 4A). The clusters that exhibited enrichment (median > 0) of ATB-associated DEGs included clusters 0, 1, 2, 5, 7, and 8, which thus represented more activated populations compared with the rest of the clusters (Figure 3C).

We then examined the expression of *IFNG* across clusters, as it was found to be one of the ATB-associated DEGs (Figure 2C), is known to be a critical cytokine involved in the TB immune response,^33^ and was produced by CD4^−^CD8^−^ γδ T cells in response to BCG stimulation (Figure 1). Clusters 0, 1, 5, and 7 had high expression of *IFNG* (Figure 4B), all of which also exhibited enrichment of ATB-associated DEGs (Figure 4A). We calculated the proportion of *IFNG*^+^ cells (*IFNG* > 1) originating from each cluster and compared that with the proportion of each cluster within the total cell population (Figure 4C). Clusters 0, 1, and 5 were found at significantly higher proportions within the *IFNG*^+^ population than expected within the total dataset, with over 50% of the *IFNG*^+^ cells originating from clusters 0 and 1.

Together, these data identify clusters 0, 1, 5, and 7 as the likely source of *IFNG*-producing CD4^−^CD8^−^ γδ T cells in ATB samples at diagnosis.

### Multiple clusters are associated with enrichment in ATB at diagnosis

Next, we investigated whether differences across the cohorts existed in the four clusters with increased ATB module scores and *IFNG* expression to highlight subsets potentially involved in responding to TB disease. We performed single-cell GSEA for each cluster with or without increased ATB module scores and *IFNG* expression. GSEA scores were highly correlated between geographical locations included in this study, suggesting minimal differences due to different environmental conditions (Figure S7A). Clusters 1 and 5 each showed similar levels of enrichment between ATB at diagnosis and healthy cohorts, while the IGRA+ cohort in cluster 7 showed significant enrichment for many pathways compared with ATB at diagnosis (Figure 4D). Although most clusters exhibited high enrichment in interferon response pathways in ATB compared with healthy cohorts, suggesting these cells may be responding to infection, clusters 0 through 4 exhibited an enrichment pattern similar to that of the global analysis of all CD4^−^CD8^−^ γδ T cells with high enrichment of many of the same pathways in ATB at diagnosis compared with healthy cohorts (Figures 4D and S7B). Many pathways were enriched in ATB at diagnosis in clusters 0 and 1, with cluster 1 separating IGRA− from IGRA+ and ATB (Figure 4D). Cluster 5 showed overlap in enrichment between ATB at diagnosis and IGRA− cohorts, while cluster 7 exhibited distinct signatures across all three cohorts (Figure 4D). When we compared the average *Z* scores of each pathway between each of the four clusters of interest and the overall dataset, we found that cluster 1 had the strongest and most significant correlation (Figure 4E), indicating an enrichment profile that most closely mirrors that of the overall dataset compared with the other clusters. Clusters 0 and 7 also had significant correlation to the overall dataset (Figure 4E). However, only cluster 0 was enriched in ATB at diagnosis in terms of frequency (Figures 3D and S6C). Overall, these data suggest that multiple clusters are associated with enrichment in ATB at diagnosis in terms of pathway enrichment and may be involved in the anti-TB immune response.

### Vδ1 and Vδ3 subsets were enriched in ATB at diagnosis

**Figure 5:**
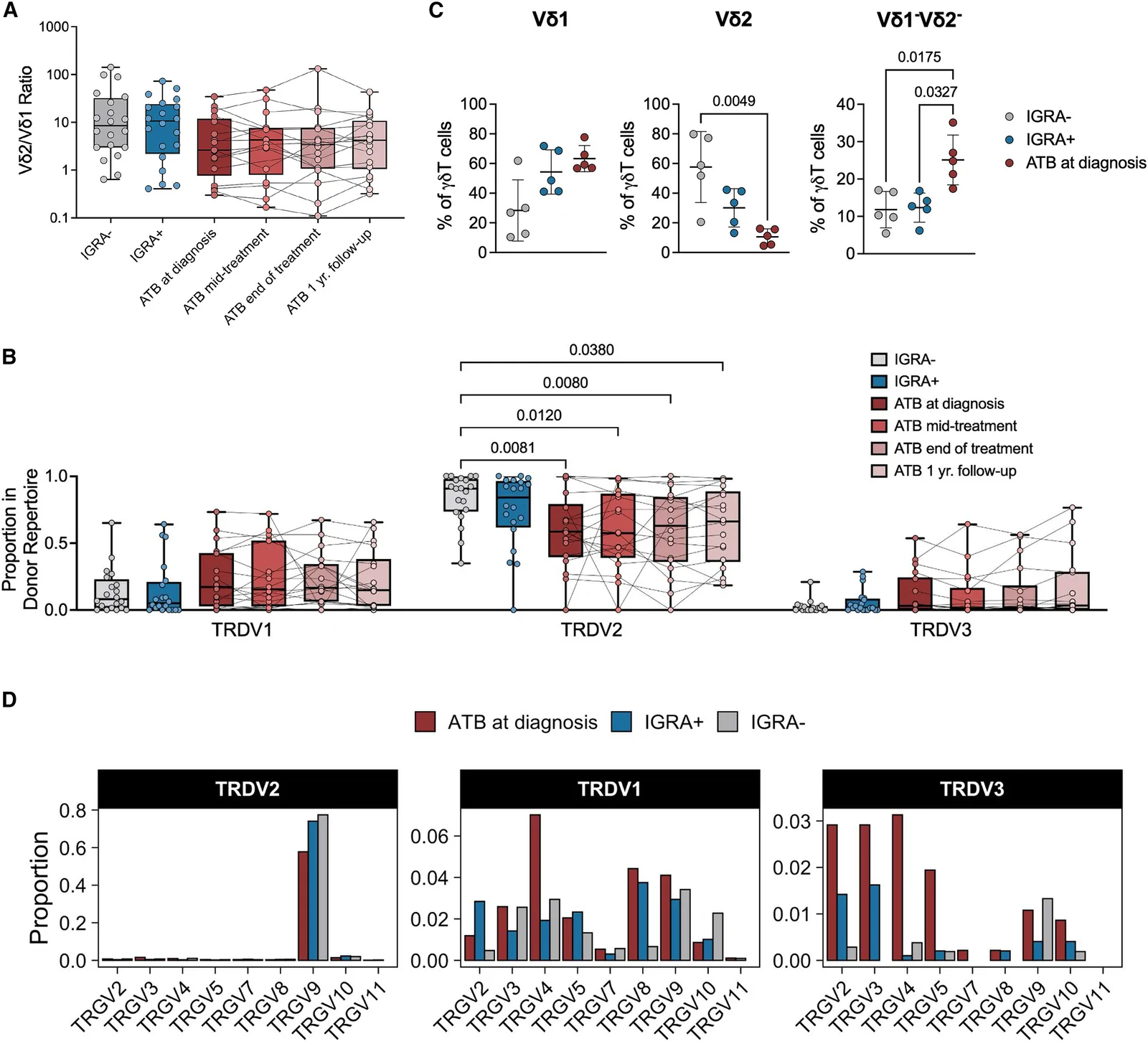
Vδ1 and Vδ3 subsets were enriched in ATB at diagnosis (A) Vδ2:Vδ1 ratio calculated for each cohort using expression of *TRDV1* and *TRDV2*. (B) Proportion of the total donor TCR repertoire expressing *TRDV1*, *TRDV2*, or *TRDV3* in IGRA−, IGRA+, and ATB cohorts across treatment timepoints for each donor. Ordinary two-way ANOVA with Tukey’s multiple comparisons test. (C) Frequency of Vδ1, Vδ2, and Vδ1^−^Vδ2^−^ T cells within the γδ T cell population in IGRA−, IGRA+, and ATB at diagnosis cohorts using flow cytometry. Data are represented as mead with SD. Kruskal-Wallis test with Dunn’s multiple comparisons test within each subset. (D) Proportion of each cohort with specific *TRGV* gene pairings for *TRDV1*, *TRDV2*, and *TRDV3*. See also Figure S8.

We next analyzed expression of TCR δ genes to determine whether there was a preference for specific gene usage between the ATB and healthy cohorts. First, we calculated the Vδ2:Vδ1 ratio per sample using the expression level of *TRDV2* and *TRDV1* genes and found a trend for lower ratios in ATB compared with healthy cohorts that persisted through the last timepoint post-treatment (Figure 5A), supporting previous findings.^11^^,^^12^ Consistent with this finding, we also noted an increase in the proportion of cells having nonzero expression of *TRDV1* and *TRDV3* and a corresponding decrease in cells expressing *TRDV2* across ATB cohorts compared with healthy individuals (Figure 5B). To further validate these findings, we used flow cytometry to measure the frequency of γδ T cell subsets. Since there is no commercially available Vδ3 antibody, we used Vδ1^−^Vδ2^−^ cells within the γδ T cell population as a proxy for Vδ3 cells as they are the next most abundant γδ T cell subset after Vδ1 and Vδ2 cells.^34^ We found a significant increase in Vδ1^−^Vδ2^−^ cell frequency in ATB at diagnosis compared with both IGRA− and IGRA+ cohorts (Figure 5C). We also found a trend for increased Vδ1 and significantly decreased Vδ2 cell frequencies in ATB at diagnosis compared with the IGRA− cohort (Figure 5C).

Next, we examined the usage of specific TCR gamma and delta gene pairs to potentially identify combinations associated with TB disease. We focused on the healthy and ATB at diagnosis cohorts, as these displayed the most striking differences in overall phenotypes. We utilized expression values of TCR variable (V) genes and only included cells expressing both TRGV and TRDV genes in this analysis. We found that the *TRDV2* TCRs nearly exclusively pair with *TRGV9* in all disease conditions, as previously described^9^ (Figure 5D). For *TRDV1* and *TRDV3* TCRs, gamma chain usage was diverse and varied across the cohorts. The increase of *TRDV1* usage in ATB at diagnosis was associated specifically with the *TRGV4* gamma chain, which was also substantially increased in combination with *TRDV3* (Figure 5D). *TRGV4*/*TRDV1* usage was increased at the mid-treatment timepoint in 8 of 17 samples (Figure S8A), while *TRGV4*/*TRDV3* usage remained relatively stable across treatment timepoints (Figure S8B). Furthermore, *TRGV4*/*TRDV1* TCRs were predominantly found within cluster 6 (naive Vδ1) in healthy cohorts and cluster 0 (cytotoxic NK-like Vδ1/3) in ATB cohorts (Figure S8C). This may indicate that the *TRGV4*/*TRDV1* gene pair is naive at steady state and becomes activated and develops a cytotoxic phenotype in ATB.^35^ Overall, these data suggest that specific γδ TCR chain pairings are associated with TB disease.

## Discussion

In this study, we characterized the *ex vivo* frequencies and transcriptional profiles of CD4^−^CD8^−^ γδ T cells across healthy and ATB cohorts and timepoints throughout anti-TB treatment. We found that γδ T cells were the primary producers of IFNγ in response to BCG (but not MTB300 peptide pool, as expected) within the CD4^−^CD8^−^ T cell population, supporting previous studies.^14^^,^^16^ Despite stable *ex vivo* frequencies across cohorts and timepoints, CD4^−^CD8^−^ γδ T cells exhibited strong transcriptional differences in ATB *ex vivo*, suggesting functional reprogramming and a bias toward specific γδ T cell subsets in response to *Mtb* infection and/or anti-TB treatment.

Using gene set enrichment analysis, we found that CD4^−^CD8^−^ γδ T cells from ATB patients were enriched for many immune activation and metabolic pathways. The enrichment of interferon alpha/gamma response pathways in ATB is consistent with previous findings confirming the involvement of these pathways in the TB immune response.^36^^,^^37^^,^^38^^,^^39^ The metabolic pathways (e.g., oxidative phosphorylation and glycolysis) that were also upregulated combined features of previously described IL-17- and IFNγ-producing γδ T cells, which are associated with tumor growth/metastasis and tumor surveillance, respectively.^40^^,^^41^^,^^42^ While the previous studies were focused on γδ T cells in cancer, IL-17 and IFNγ have also been implicated in TB immunity, of which γδ T cells may be an important source.^2^^,^^43^^,^^44^^,^^45^^,^^46^ Additionally, CD4^−^CD8^−^ γδ T cells from ATB showed increased expression of genes related to activation and cytotoxicity (*CD69*, *IFNG*, and *GZMB*), suggesting that CD4^−^CD8^−^ γδ T cells with an effector phenotype are involved in *Mtb* immune responses.

We identified four clusters (0, 1, 5, and 7) as clusters of interest due to their high ATB gene module scores and *IFNG* expression, suggesting that these subsets may be involved in anti-TB immunity. The gene set enrichment patterns across cohorts in these clusters suggest that TB disease is associated with heterogeneous modulation of immune pathways across distinct γδ T cell subsets, reflecting potential differences in baseline functional programming, antigen exposure history, or sensitivity to environmental cues. Cluster 0 (cytotoxic NK-like Vδ1/3) had the highest proportion of Vδ3 cells and was the only cluster that was significantly enriched in ATB at diagnosis compared with healthy cohorts. We also found a decreased Vδ2:Vδ1 ratio and increased frequency of Vδ3 cells in the circulation of TB patients. This was a striking observation, as Vδ1 and Vδ3 cells are more commonly found in tissues compared with circulation and may suggest a role for these subsets in TB.^8^^,^^11^^,^^12^ Other studies have revealed that circulating Vδ1 and Vδ3 cells increase in frequency in other contexts such as cancer and viral infections and have suggested that these cells may be involved in memory formation and antiviral immunity.^47^^,^^48^^,^^49^ However, while γδ T cells may respond to *Mtb* antigens, the lack of substantial differences in frequency of γδ T cell subsets observed throughout treatment timepoints underscore that this response alone is insufficient for disease clearance. Our findings indicate that these two Vδ subsets may also play a role in anti-TB immunity.

Additionally, cluster 0, which we annotated as a cytotoxic NK-like Vδ1/3 subset, closely resembles a previously defined CD8^+^ NK-like γδ T cell population in individuals with persistent *Mtb* infection.^50^ This population was found to consist mostly of *TRDV1*-expressing cells and exhibited significantly decreased ability for TCR-mediated activation with a strong capacity for CD16-mediated cytotoxicity. Thus, cluster 0 cells may also be activated through non-TCR-mediated mechanisms such as antibody-dependent cellular cytotoxicity. γδ T cells with NK-like phenotypes have also been found to be enriched in other acute and chronic contexts, suggesting that this subset is involved in the immune response in various conditions.^50^^,^^51^^,^^52^^,^^53^ Furthermore, a recent study revealed a cytotoxic effector phenotype in Vδ1/3 cells following intravenous BCG vaccination that protects against TB.^54^ These pro-inflammatory responses were found in Vδ1/3 cells isolated from both the blood and airway of nonhuman primates after stimulation with *Mtb* lysate, but *Mtb*-reactive cells were enriched in the lung. As this cell population closely aligns with our cytotoxic NK-like Vδ1/3 cluster in its overall transcriptomic profile, our findings raise the possibility that our subset may similarly be enriched in the human lung during and protect against active disease.

We observed a decrease in the Vδ2:Vδ1 ratio in ATB compared with healthy cohorts, supporting previous findings.^11^^,^^12^ Along with this, there was an increase in *TRDV3* and decrease in *TRDV2* usage at the gene level across ATB timepoints and at the protein level in ATB at diagnosis in comparison with healthy cohorts. Further analysis revealed a diverse TCR gene pair repertoire. Both *TRDV1* and *TRDV3* gene pairs associated with ATB included *TRGV4*, which has been found at higher frequencies in the blood of TB patients,^35^ suggesting potential involvement in the anti-TB immune response. Although Vδ3 cells are rarely found in the blood of healthy individuals, higher frequencies in the circulation have been reported in the context of cancer and viral infections.^55^ Notably, Vδ3 cells recognize MR1-and CD1d-presented antigens and possess cytotoxic and cytokine-producing functions, suggesting a role in antimicrobial activity.^56^^,^^57^ Together with the recent evidence demonstrating protective responses by Vδ1/3 subsets following BCG vaccination,^54^ our results corroborate the idea that Vδ1/3 cells may represent a previously underappreciated component of the immune response to *Mtb*.

All four timepoints analyzed in the ATB cohort exhibited transcriptomic profiles distinct from those of healthy individuals. Additionally, the bias for Vδ1 and Vδ3 cells was present throughout all four timepoints. This is remarkable, as the fourth timepoint corresponds to six months after the end of anti-TB therapy, approximately one-year post-diagnosis. Together, these results suggest that *Mtb* infection and/or anti-TB treatment induce long-lasting transcriptomic changes in CD4^−^CD8^−^ γδ T cells. Durable transcriptomic differences between healthy and ATB follow-up cohorts have been shown previously, though the number of DEGs gradually decreased compared with that of healthy vs. ATB at diagnosis, and these data were obtained from whole blood.^58^ It remains to be defined whether these changes are only long-lasting in specific subsets, such as CD4^−^CD8^−^ γδ T cells as described here, or systemic. Additionally, studies have reported both increased and decreased lipid antigen-specific T cell responses after starting anti-TB therapy, indicating potential changes in γδ T cell responses as they can recognize lipid antigens.^59^^,^^60^^,^^61^ As the conventional TB treatment regimen of isoniazid, rifampicin, pyrazinamide, and ethambutol is known to be bactericidal, the resulting antigen availability may be altered and lead to modified unconventional T cell responses as shown here.^62^

In conclusion, our study reveals long-lasting transcriptomic changes in CD4^−^CD8^−^ γδ T cells in ATB compared with healthy individuals. We also identify a distinct population of CD4^−^CD8^−^ γδ T cells, cytotoxic NK-like Vδ1/3 cells, that is enriched in ATB at diagnosis in terms of frequency, gene module scores, and pathway enrichment. While Vδ3 cells were significantly more associated with TB disease, *TRGV4*/*TRDV1*-expressing T cells may be recognizing *Mtb* antigens and contributing to the cytotoxic profile seen in cluster 0. These findings further contribute to our understanding of unconventional T cell responses in TB and highlight specific γδ T cell populations as potentially important, yet understudied, components of the *Mtb* immune response.

### Limitations of the study

Our study has several limitations. Our analysis was restricted to circulating γδ T cells and does not directly assess the responses occurring in the lung during TB infection, which may be different. Additionally, whether and how the different γδ T cell clusters herein described recognize *Mtb* and how this may differ across TB disease states were not addressed by the present study. Functionally validating the effector potential of these subsets, particularly Vδ3 cells, to determine whether they contribute to host protection, immunopathology, or both would be crucial to better understanding the roles of γδ T cells in the *Mtb* immune response. Further work using targeted TCRγδ sequencing could reveal additional information on specific CDR3 sequences associated with TB disease and lead to the identification of putative ligands.

## Resource availability

### Lead contact

Requests for further information and resources should be directed to and will be fulfilled by the lead contact, Bjoern Peters (bpeters@lji.org).

### Materials availability

This study did not generate new unique reagents.

### Data and code availability

• De-identified human data used in this publication can be found at Gene Expression Omnibus: GSE312606 for single-cell RNA sequencing data and ImmPort: IS SDY3462 for flow cytometry data.
• This paper does not report original code.
• Any additional information required to reanalyze the data reported in this paper is available from the lead contact upon request.

## Acknowledgments

We thank the Flow Cytometry Core and Sequencing Core Facility at the La Jolla Institute for Immunology for their valued role in sample acquisition and sequencing. We acknowledge support from the 10.13039/100000002National Institutes of Health grant U19 AI118626 (to B.P.) and 10.13039/100000002National Institutes of Health contract 75N93024C00057 (to B.P. and C.S.L.A).

## Author contributions

J.G.B., C.S.L.A, and B.P. participated in the design and direction of the study. K.K. and R.T. performed and analyzed the experiments. K.K. performed bioinformatic analysis. A.C., J.G., and R.T. assisted in bioinformatic analysis. R.T., J.P., H.G., D.D.S., W.G., A.D.D.S., and T.J.S. recruited participants, performed clinical evaluations, and isolated PBMCs. K.K., J.G.B., C.S.L.A., and B.P. wrote the manuscript. All authors read, edited, and approved the manuscript before submission.

## Declaration of interests

The authors declare no competing interests.

## STAR★Methods

### Key resources table

**Table:** 

REAGENT or RESOURCE	SOURCE	IDENTIFIER
**Antibodies**		

Mouse anti-human CD3 AF700 (clone UCHT1)	Waters Biosciences	Cat#: 557943; RRID: AB_396952
Mouse anti-human CD4 APC-eFluor 780 (clone RPA-T4)	eBioscience	Cat#: 47004942; RRID: AB_1272044
Mouse anti-human CD8a BV650 (clone RPA-T8)	BioLegend	Cat#: 301042; RRID: AB_2563505
Mouse anti-human CD161 APC (clone HP-3G10)	eBioscience	Cat#: 17161942; RRID: AB_10597894
Mouse anti-human TCR Vα7.2 PE/Cy7 (clone 3C10)	BioLegend	Cat#: 351712; RRID: AB_2561994
Mouse anti-human TCR γ/δ PE (clone 11F2)	Waters Biosciences	Cat#: 347907; RRID: AB_400359
Mouse anti-human CCR7 PerCP/Cy5.5 (clone G043H7)	BioLegend	Cat#: 353220; RRID: AB_10916121
Mouse anti-human CD45RA eFluor450 (HI100)	eBioscience	Cat#: 48045842; RRID: AB_1272059
Mouse anti-human CD14 V500 (clone M5E2)	Waters Biosciences	Cat#: 561391; RRID: AB_10611856
Mouse anti-human CD19 V500 (clone HIB19)	Waters Biosciences	Cat#: 561121; RRID: AB_10562391
Mouse anti-human IFN gamma FITC (clone 4S.B3)	Invitrogen	Cat#: 11-7319-82; RRID: AB_465415
TotalSeq-C0159 mouse anti-human HLA-DR (clone L243)	BioLegend	Cat#: 307663; RRID: AB_2800795
TotalSeq-C0045 mouse anti-human CD4 (clone SK3)	BioLegend	Cat#: 344651; RRID: AB_2800921
Mouse anti-human CCR7 BB515 (clone 2-L1-A)	Waters Biosciences	Cat#: 566765; RRID: AB_2869855
Mouse anti-human CD11c BV785 (clone 3.9)	BioLegend	Cat#: 301644; RRID: AB_2565779
Mouse anti-human CD123 PerCP/Cy5.5 (clone 6H6)	BioLegend	Cat#: 306016; RRID: AB_2264693
Mouse anti-human CD14 BV480 (clone M5E2)	Waters Biosciences	Cat#: 746304; RRID: AB_2743629
Mouse anti-human CD16 BUV737 (clone 3G8)	Waters Biosciences	Cat#: 612787; RRID: AB_2870114
Mouse anti-human CD19 BV605 (clone HIB19)	BioLegend	Cat#: 302244; RRID: AB_2562015
Mouse anti-human CD1c BV650 (clone L161)	BioLegend	Cat#: 331542; RRID: AB_2800866
Mouse anti-human CD20 BUV563 (clone 2H7)	Waters Biosciences	Cat#: 748456; RRID: AB_2872872
Mouse anti-human CD3 BUV805 (clone UCHT1)	Waters Biosciences	Cat#: 612895; RRID: AB_2870183
Mouse anti-human CD38 PE/Dazzle 594 (clone HIT2)	BioLegend	Cat#: 303538; RRID: AB_2564105
Mouse anti-human CD45 BUV395 (clone HI30)	Waters Biosciences	Cat#: 563791; RRID: AB_2869519
Mouse anti-human CD45RA BV570 (clone HI100)	BioLegend	Cat#: 304132; RRID: AB_2563813
Mouse anti-human CD56 APC (clone 5.1H11)	BioLegend	Cat#: 362504; RRID: AB_2563913
Mouse anti-human CD71 PE-Cy5	Waters Biosciences	Cat#: 551143; RRID: AB_394071
Mouse anti-human CD8 BUV496 (clone RPA-T8)	Waters Biosciences	Cat#: 612942; RRID: AB_2870223
Mouse anti-human HLA-DR PE/Cy7 (clone LN3)	BioLegend	Cat#: 327018; RRID: AB_2566389
Mouse anti-human IgD BV750 (clone IA6-2)	Waters Biosciences	Cat#: 747484; RRID: AB_2868411
Mouse anti-human TCR γ/δ BV421 (clone 11F2)	Waters Biosciences	Cat#: 666657
Mouse anti-human CD107a BB700 (clone H4A3)	Waters Biosciences	Cat#: 566558; RRID: AB_2869782
Mouse anti-human CD137 BUV395 (clone 4B4-1)	Waters Biosciences	Cat#: 745737; RRID: AB_2743209
Mouse anti-human TCR γ/δ purified (clone 11F2)	Waters Biosciences	Cat#: 347900; RRID: AB_400356
Goat anti-mouse IgG PE (clone Poly4053)	BioLegend	Cat#: 405307; RRID: AB_315010
Mouse anti-human CD14 PE/Fire 810 (clone S18004B)	BioLegend	Cat#: 399223; RRID: AB_2936545
Mouse anti-human CD19 BUV805 (clone HIB19)	Waters Biosciences	Cat#: 568331; RRID: AB_3684187
Mouse anti-human CD3 APC-R700 (clone UCHT1)	Waters Biosciences	Cat#: 565119; RRID: AB_2744385
Mouse anti-human TCR V delta 1 FITC (clone TS8.2)	Invitrogen	Cat#: TCR2730; RRID: AB_223624
Mouse anti-human TCR Vδ2 APC (clone B6)	BioLegend	Cat#: 331418; RRID: AB_2687324
Mouse anti-human TCR Vα24-Jα18 PE/Dazzle 594 (clone 6B11)	BioLegend	Cat#: 342920; RRID: AB_2566312
Mouse anti-human TCR Vα7.2 PE/Cy7 (clone 3C10)	BioLegend	Cat#: 351712; RRID: AB_2561994
Mouse anti-human CD161 BV421 (clone HP-3G10)	BioLegend	Cat#: 339914; RRID: AB_2561421
Mouse anti-human CD26 BV510 (clone L272)	Waters Biosciences	Cat#: 745067; RRID: AB_2742687
Mouse anti-human CD16 RB545 (clone 3G8)	Waters Biosciences	Cat#: 569242; RRID: AB_3684898
Mouse anti-human CD56 PerCP-eFluor 710 (clone CMSSB)	Invitrogen	Cat#: 46-0567-42; RRID: AB_10548939
Mouse anti-human TCRαβ BUV563 (clone IP26)	Waters Biosciences	Cat#: 749197; RRID: AB_2873575
Mouse anti-human CD69 BV605 (clone FN50)	Waters Biosciences	Cat#: 562989; RRID: AB_2737935
Mouse anti-human IFNγ RB780 (clone B27)	Waters Biosciences	Cat#: 568686; RRID: AB_3684468
Rat anti-human and viral IL-10 BV711 (clone JES3-9D7)	Waters Biosciences	Cat#: 564050; RRID: AB_2738564
Mouse anti-human IL-17A BV786 (clone N49-653)	Waters Biosciences	Cat#: 563745; RRID: AB_2738401
Rat anti-human IL-2 BUV737 (clone MQ1-17H12)	Waters Biosciences	Cat#: 612836; RRID: AB_2870158
Mouse anti-human TNF alpha PerCP-Cy5.5 (clone Mab11)	Invitrogen	Cat#: 45-7349-42; RRID: AB_10718247
Mouse anti-human granulysin AF647 (clone DH2)	BioLegend	Cat#: 348006; RRID: AB_2110110
Mouse anti-human/mouse granzyme B Pacific Blue (clone GB11)	BioLegend	Cat#: 515408; RRID: AB_2562196

**Biological samples**		

*M. bovis* BCG-Danish	R. Mortensen, SSI, Denmark	N/A
*M. bovis* Pasteur	D. Barber, NIH, USA	N/A
Mtb H37Rv whole cell lysate	BEI Resources	Cat#: NR-14822

**Chemicals, peptides, and recombinant proteins**		

MTB300 peptide pool	C.S. Lindestam Arlehamn/B. Peters, LJI, USA	N/A
Fixable viability dye eFluor 506	eBioscience	Cat#: 65-0866-14
Zombie UV Fixable Viability Kit	BioLegend	Cat#: 423107
BD Horizon Brilliant Stain Buffer Plus	Waters Biosciences	Cat#: 566385

**Critical commercial assays**		

Cyto-Fast Fix/Perm Buffer Set	BioLegend	Cat#: 426803

**Deposited data**		

10× sequencing data	This paper	GEO: GSE312606
Flow cytometry data	This paper	ImmPort: IS SDY3462

**Software and algorithms**		

R v4.4.2	R foundation	https://www.r-project.org/
Seurat v4.4.0	Satija Lab	https://satijalab.org/seurat/
FlowJo v10	Waters Corporation	https://www.flowjo.com/
GraphPad Prism v10.6.0	GraphPad Software	https://www.graphpad.com/

### Experimental model and study participant details

Participants were from South Africa, Sri Lanka, United States of America, and Peru. Samples were age- and sex-matched across the sites. The individuals were segregated into three main cohorts: active TB (ATB, *n* = 40), IGRA+ (*n* = 56), and IGRA− (*n* = 25); additional information can be found in Figure 2A and Table S6. The healthy IGRA+ cohort was identified as positive for the IFNγ-release assay (IGRA; QuantiFERON-TB Gold In-Tube, Cellestis or T-SPOT.TB, Oxford Immunotec) with the absence of symptoms consistent with TB or other clinical or radiographic signs of ATB. ATB was defined, in the absence of any other significant co-morbidity, as 1) presence of clinical symptoms and/or radiological/histological evidence of pulmonary TB and 2) microbiologically confirmed by sputum or *Mtb* culture. ATB individuals underwent the standard anti-TB regimen for drug-susceptible TB consisting of an intensive phase of two months with isoniazid, rifampin, pyrazinamide, and ethambutol followed by a continuation phase of four months with isoniazid and rifampin. IGRA-individuals had no past medical history of TB or exposure to *Mtb* or evidence of *Mtb*-sensitization as confirmed by a negative IFNγ-release assay. All participants were confirmed negative for HIV. Both male and female participants were included in this study. The potential influence of sex assigned at birth on the measured immunological outcomes was assessed, and no significant sex-associated differences were detected. Accordingly, data from male and female participants were analyzed together.

#### Ethics statement

Human study participants were enrolled at the National Hospital for Respiratory Diseases, Welisara (Sri Lanka), South African Tuberculosis Vaccine Initiative, Western Cape Province (South Africa), University of California San Diego Anti-Viral Research Center (United States of America), or Universidad Peruana Cayetano Heredia (Peru). Ethical approval was maintained through the La Jolla Institute for Immunology Institutional Review Board (IRB) or the Human Research Ethics Committee of the University of Cape Town. The University of Colombo Ethics Review Committee served as the National Institute of Health registered IRB for the Kotelawala Defense University. All clinical investigations were conducted according to the principles expressed in the Declaration of Helsinki, and all participants provided written informed consent prior to participation in the study.

### Method details

#### PBMC thawing

PBMCs were obtained by density gradient centrifugation (Ficoll-Hypaque, Amersham Biosciences/GE Healthcare) from leukapheresis or whole-blood samples, according to the manufacturer’s instructions. Cells were resuspended at 50–100 million cells per milliliter in FBS (Gemini Bio-Products) containing 10% DMSO (Sigma) and cryopreserved in liquid nitrogen. Cryopreserved PBMCs were quickly thawed by incubating each cryovial at 37°C for 1–2 min. Cells were then transferred into 9 mL of cold HR5 medium (RPMI 1640 with L-Glutamin and 25 mM HEPES [Omega Scientific], supplemented with 5% human AB serum [GemCell], 1% Penicillin/Streptomycin [Gibco], and 1% Glutamax [Gibco]) and 20 U/mL Benzonase Nuclease (Millipore). Cells were centrifuged and resuspended in medium to determine cell concentration and viability using trypan blue and a hemacytometer.

#### Intracellular cytokine staining

1 × 10^6^ thawed PBMCs were plated per well in 96-well round-bottom plates in HR5 medium. Cells were stimulated with *M. bovis* BCG-Danish (BCG vaccine SSI lyophilized powder for injection resuspended in provided diluent and stored at 4°C, *M. bovis* BCG Danish strain 1331; gifted from R. Mortensen, SSI, Denmark) or *M. bovis* Pasteur (frozen stocks at −80°C in 7H9 media, thawed, washed and resuspended in PBS; gifted from D. Barber, NIH, USA) at 100 μg/mL or 50 μg/mL (2–8 × 10^6^ bacteria/mL), or MTB300 (2 μg/mL per peptide); anti-CD28 (1 μg/mL) and anti-CD49d (1 μg/mL) were present in all stimuli preparations. The plate was incubated for 5 h at 37°C in a 5% CO_2_ incubator. BFA and monensin were added to each well per manufacturer’s recommended protocol and the plate was further incubated at 37°C for an additional 7 h and then kept at 4°C until staining. Cells were stained with a surface panel containing anti-CD3 Alexa Fluor 700 (Waters Biosciences, 557943), anti-CD4 APC-eFluor780 (eBioscience, 47004942), anti-CD8 brilliant violet 650 (BV650; BioLegend, 301042), anti-CD161 APC (eBioscience, 17161942), anti-TCR Vα7.2 phycoerythrin (PE)-Cy7 (BioLegend, 351712), anti-pan γδTCR PE (Waters Biosciences, 347907), anti-CCR7 PerCP-Cy5.5 (BioLegend, 353220), anti-CD45RA eFluor450 (eBioscience, 48045842), anti-CD14 V500 (Waters Biosciences, 561391), anti-CD19 V500 (Waters Biosciences, 561121), and fixable viability dye eFluor 506 (eBioscience, 65-0866-14). Cells were fixed with a 4% paraformaldehyde solution for 10 min, permeabilized for 10 min in saponin buffer (0.2 g saponin, 400 μL sodium acetate, 4 mL BSA, in PBS to 40 mL total volume) and blocked with 10% FBS for 5 min on ice. Samples were then stained with anti-IFNγ FITC (eBioscience, 11731982) in saponin buffer for 30min at room temperature and then washed. Samples were analyzed on a BD LSR II. The gating strategy is shown in Figure S1.

#### Flow cytometry for 10× single-cell RNA sequencing

Freshly thawed PBMCs were incubated for 10 min with Zombie UV Fixable Viability Kit (BioLegend) following the manufacturer’s protocol. To neutralize and block unbound viability dye, 10% fetal bovine serum (FBS) in 1× PBS was added to the cells followed by a centrifugation step. FcR blocking reagent (BioLegend) at a 1:50 dilution from the stock reagent solution was added to the cells. Samples were further incubated for 10 min at room temperature. For oligonucleotide-conjugated antibody staining, 2 μL of each TotalSeq™-C antibody was added per sample in a final staining volume not exceeding 100 μL in 1× PBS and cells were incubated at room temperature for 15 min. TotalSeq™-C antibodies that were used included anti-HLA-DR (clone L243, barcode 0159) and anti-CD4 (clone SK3, barcode 0045), as well as several anti-CD45 or anti-CD298 plus anti-β2-microglobulin antibodies to identify each sample in downstream analyses.

Samples were spun down and excess unbound antibody was removed before proceeding to the next step. A cocktail of fluorochrome-conjugated antibodies, 10 μL BD Horizon™ Brilliant Stain Buffer Plus (Waters Biosciences), and 1× PBS were added to a final volume of 100 μL. The antibodies included in the cocktail were anti-CCR7 BB515 (Waters Biosciences, clone 2-L1-A), anti-CD11c BV785 (BioLegend, clone 3.9), anti-CD123 PerCP-Cy5.5 (BioLegend, clone 6H6), anti-CD14 BV480 (Waters Biosciences, clone M5E2), anti-CD16 BUV737 (Waters Biosciences, clone 3G8), anti-CD19 BV605 (BioLegend, clone HIB19), anti-CD1c BV650 (BioLegend, clone L161), anti-CD20 BUV563 (Waters Biosciences, clone 2H7), anti-CD3 BUV805 (Waters Biosciences, clone UCHT1), anti-CD38 PE-Dazzle594 (BioLegend, clone HIT2), anti-CD4 APC-eF780 (eBiosciences, clone RPA-T4), anti-CD45 BUV395 (Waters Biosciences, clone HI30), anti-CD45RA BV570 (BioLegend, clone HI100), anti-CD56 APC (BioLegend, clone 5.1H11), anti-CD71 PE-Cy5 (Waters Biosciences, clone M-A712), anti-CD8 BUV496 (Waters Biosciences, clone RPA-T8), anti-HLA-DR PE-Cy7 (BioLegend, clone LN3), anti-IgD BV750 (Waters Biosciences, clone IA6-2), and anti-TCRγδ BV421 (Waters Biosciences, clone 11F2). Cells were incubated for 15 min in the dark at room temperature. Cells were washed once in 10 mL of 1× PBS to remove all unbound antibody and stored at 4°C protected from light for up to 4 h until flow cytometry acquisition. Live singlets CD3^+^CD19^−^CD14^−^CD8^−^ cells were sorted using a BD Symphony S6 sorter and sequenced using droplet-based 10× Genomics (Figure S3).

#### Single-cell RNA sequencing data preparation

The number of cells sorted per sample ranged from 241 to 3,333 depending on the quality of each sample. Sorted cells were resuspended at a concentration of 1,800 cells per μL in ice-cold PBS with 0.04% BSA. Single-cell suspensions were then immediately loaded on the 10× Genomics Chromium Controller with a loading target of 30,000–60,000 cells. Libraries were generated using the Chromium Next GEM Single Cell 5′ Reagent Kit v2 (Dual Index) per the manufacturer’s instructions. Additional steps were included for the amplification of HTO barcodes and V(D)J libraries (10× Genomics). Libraries were sequenced on an Illumina NovaSeq with a target of 30,000 reads per cell RNA library, 5,000 reads per cell HTO barcode library, and 5,000 reads per cell for V(D)J libraries. As the V(D)J libraries utilized the default TCRαβ primers, the data was not used in this work but were analyzed further in another study.^63^

#### scRNA-seq analysis – Hashtag demultiplexing, quality control, γδTCR isolation, clustering

Libraries were demultiplexed and FASTQ files were created using CellRanger *mkfastq* and mapped with the CellRanger *multi* pipeline (v5.0.0)^64^ against reference genome GRCh38 (2020-A). TCR genes belonging to TCR α, β, γ, or δ from the GEX matrix were aggregated to prevent clusters forming based on individual TCR gene expression. The GEX and HTO/ADT data were aggregated to form a Seurat object using the R package Seurat (v4.4.0)^32^ and quality control metrics (mitochondrial percent <8, nFeature_RNA ≥1,000, nCount_RNA <15,000, HTO singlets) and assay normalizations (*SCTransform* for GEX, CLT for HTO/ADT) were applied. The object was integrated using the *IntegrateData* function with the SCT normalization method.

Cells within the Seurat object that had expression of both aggregated TCR γ and δ genes (SCT assay) (Figure S4) and no expression of ADT-CD4 were selected to create a new Seurat object containing CD4^−^CD8^−^ γδ T cells. The new Seurat object was then subjected to dimensionality reduction and clustering using *FindNeighbors*, *RunUMAP* with 30 dimensions, and *FindClusters* with a resolution of 0.5. Cluster-specific markers were obtained using *PrepSCTFindMarkers* and *FindAllMarkers* with a min.pct of 0.25 and a logfc.threshold of 0.25. Cluster-specific markers can be found in Table S4. All visualization plots were produced using the packages Seurat, ggplot2, ComplexHeatmap, or GraphPad Prism.

#### scRNA-seq analysis – Differential expression, functional enrichment

Each sample was downsampled to 86 cells across the entire dataset for cohort-specific differential expression and gene set enrichment analyses. This threshold was chosen as the first quantile of the overall dataset and 80 of 107 samples had at least 86 cells. For cluster-specific analyses, each sample per cluster was downsampled to the median number of cells in that cluster. DEG analysis between groups was performed using the MAST algorithm^65^ on the log-normalized RNA expression matrix using Seurat’s *FindMarkers* function with a logfc.threshold of 0. To account for unequal cell numbers between groups, cells were randomly subsampled from the larger group to match the number of cells in the smaller group prior to DEG analysis. A gene was considered significantly differentially expressed if the adjusted *p*-value was 0.05 and the |log_2_ fold change| was greater than 0.3. Enrichment analyses were performed using the packages escape or fgsea with hallmark pathways. Module scores were calculated using the Seurat *AddModuleScore* function.

#### Flow cytometry for identifying γδ T cell subsets

Freshly thawed PBMC samples were rested overnight at 37°C with 5% CO_2_ in the presence of anti-CD107a BB700 (Waters Biosciences, clone H4A3) and stimulated with either 1 μg/mL anti-CD3 (Invitrogen, 16-0037-85) and 1 μg/mL anti-CD28 (Invitrogen, 16-0289-85), 0.13e6 CFU/mL *M. bovis* BCG-Pasteur (frozen stocks at −80°C in 7H9 media, thawed, washed and resuspended in PBS; gifted from D. Barber, NIH, USA), or 10 μg/mL Mtb H37Rv whole cell lysate (BEI Resources, NR-14822). 20 h later, anti-CD137 BUV395 (Waters Biosciences, clone 4B4-1) was added along with brefeldin A and monensin (Waters Biosciences) and kept at 37°C for another 4 h. At the end of incubation, cells were stained with Zombie UV (BioLegend) according to manufacturer’s recommendation. Cells were then stained with unconjugated anti-TCRγδ (Waters Biosciences, clone 11F2) for 20 min, washed, stained with secondary goat anti-mouse-Ig PE (BioLegend, clone Poly4053) for 20 min, and washed again. The rest of the surface antibodies were then added: anti-CD14 PE/Fire 810 (BioLegend, clone S18004B), anti-CD19 BUV805 (Waters Biosciences, clone HIB19), anti-CD3 APC-R700 (Waters Biosciences, clone UCHT1), anti-CD4 APC-eF780 (Invitrogen, clone RPA-T4), anti-CD8a BV650 (BioLegend, clone RPA-T8), anti-Vδ1 FITC (Invitrogen, clone TS8.2), anti-Vδ2 APC (BioLegend, clone B6), anti-Vα24-Jα18 PE/Dazzle 594 (BioLegend, clone 6B11), anti-Vα7.2 PE-Cy7 (BioLegend, clone 3C10), anti-CD161 BV421 (BioLegend, clone HP-3G10), anti-CD26 BV510 (Waters Biosciences, clone L272), anti-CD16 RB545 (Waters Biosciences, clone 3G8), anti-CD56 PerCP-eFluor710 (Invitrogen, clone CMSSB), anti-TCRαβ BUV563 (Waters Biosciences, clone IP26), anti-CD69 BV605 (Waters Biosciences, clone FN50), and anti-CD137 BUV395 again to bolster the earlier staining step. After surface staining was complete, cell membranes were rendered permeable using Cyto-Fast Fix/Perm Buffer Set (BioLegend). Intracellular staining was then performed using anti-IFNγ RB780 (Waters Biosciences, clone B27), anti-IL-10 BV711 (Waters Biosciences, clone JES3-9D7), anti-IL-17A BV786 (Waters Biosciences, clone N49-653), anti-IL-2 BUV737 (Waters Biosciences, clone MQ1-17H12), anti-TNF PerCP-Cy5.5 (Invitrogen, clone Mab11), anti-granulysin AF647 (BioLegend, clone DH2), and anti-granzyme B Pacific Blue (BioLegend, clone GB11). Samples were analyzed on a Cytek Aurora with 5L configuration. Partial data shown in Figure 5C (unstimulated).

### Quantification and statistical analysis

Statistical analyses were performed using Friedman test with Dunn’s multiple comparisons test, pairwise Wilcoxon rank-sum tests with Bejamini-Hochberg multiple testing correction, exact binomial test, Pearson correlation, permutation tests, two-way ANOVA with Tukey’s multiple comparisons test, or Kruskal-Wallis test with Dunn’s multiple comparisons test where appropriate (listed in the caption for each figure). Prism 10.6.0 (GraphPad) and R (v4.4.2) were used for these calculations. Values pertaining to significance are noted in the respective figure, and *p* < 0.05 was defined as statistically significant.

## Supplemental information

Document S1. Figures S1–S8 and Tables S1 and S5

Table S2. Gene set enrichment results including leading edge genes for IGRA−, IGRA+, and ATB at diagnosis cohorts in each cluster

Table S3. Differential gene expression results between healthy (IGRA− and IGRA+) and ATB at diagnosis cohorts

Table S4. Cluster markers identified by Seurat’s FindMarkers function

Table S6. Donor information
