# Supplementary material for: Enrichment of CD4−CD8− cytotoxic Vδ1/3 T cells in pulmonary tuberculosis disease
Source: iScience. 2026 Aug 19;29(9):117241. doi: 10.1016/j.isci.2026.117241 (PMC13521104; doi:10.1016/j.isci.2026.117241)
Supplement: Supplementary file 1 [file mmc1.pdf]

iScience, Volume *29*

Supplemental information

Enrichment of CD4^{-}CD8^{-} cytotoxic

Vδ1/3 T cells in pulmonary tuberculosis disease

Kendall Kearns, Rashmi Tippalagama, Ashu Chawla, Jason Greenbaum, Aruna Dharshan De Silva, Wathsala Gunasinghe, Judy Perera, Hansani Gunasekera, Darsha D. Senevirathne, Thomas J. Scriba, Julie G. Burel, Cecilia S. Lindestam Arlehamn, and Bjoern Peters

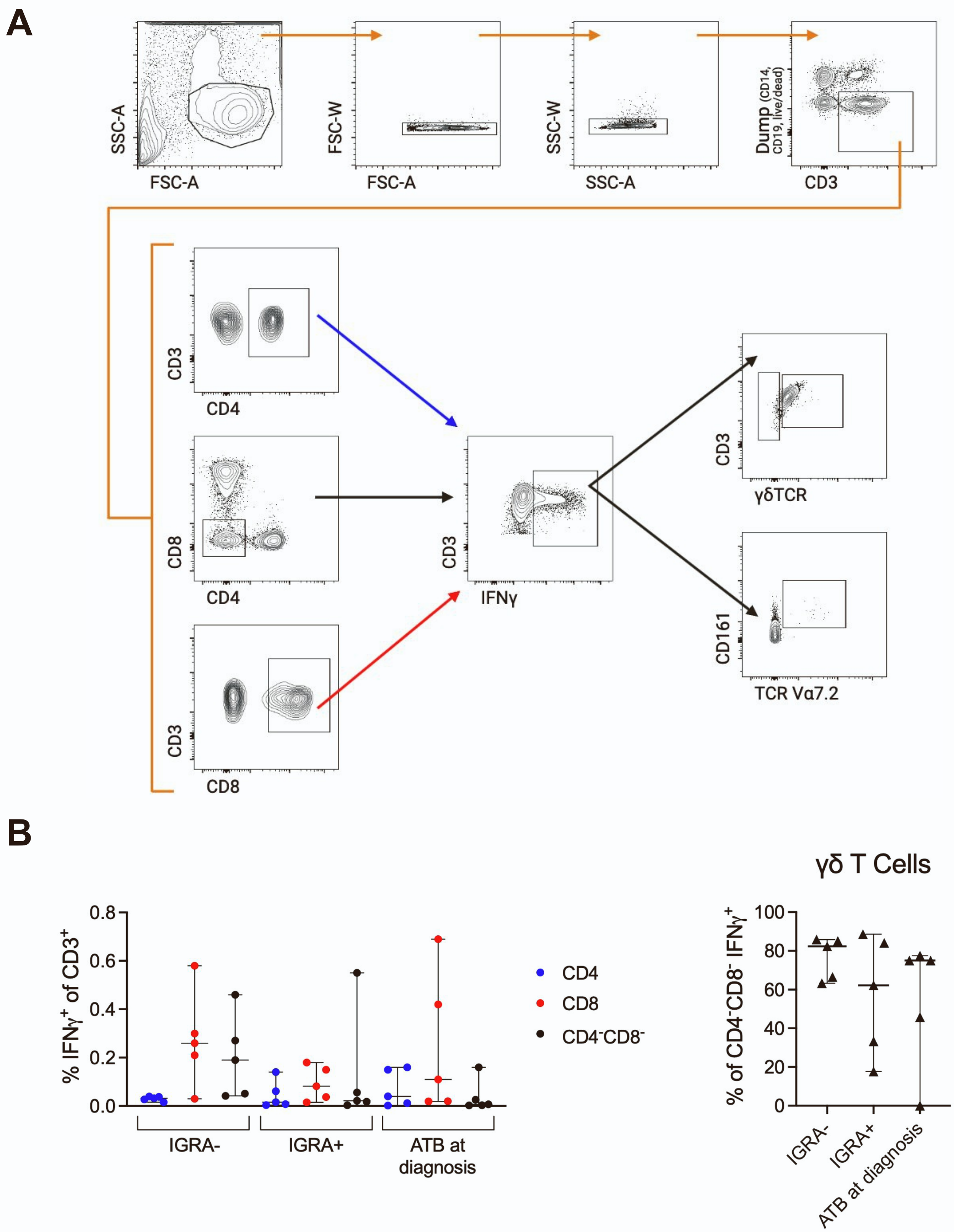

Figure S1. Flow cytometry gating strategy and IFNγ responses following BCG stimulation.

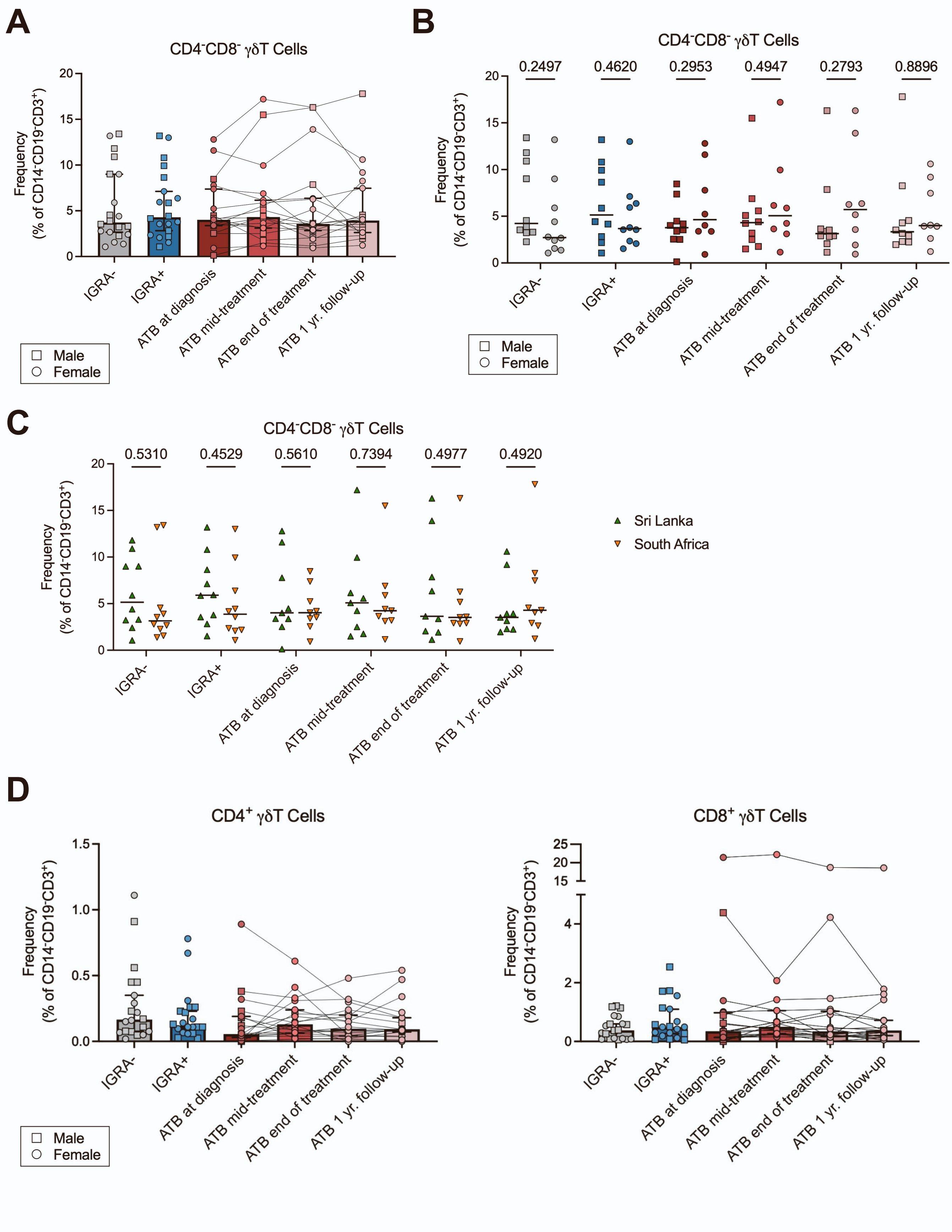

Figure S2. Flow cytometric analysis of $CD4^{-}CD8^{-}$ γδ T cell frequencies across cohorts.

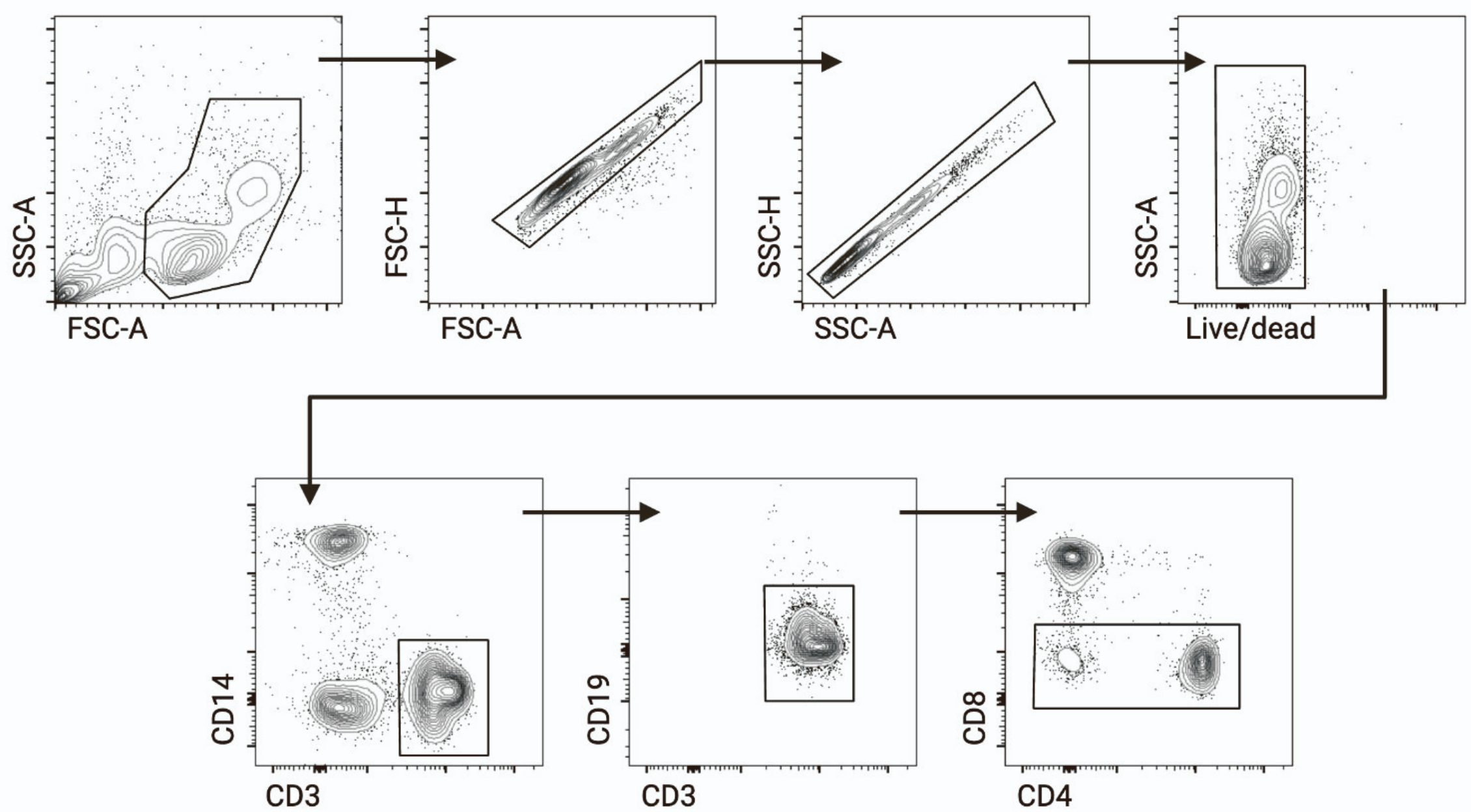

Figure S3. Gating strategy for fluorescence-activated sort of CD8⁻ T cells for single-cell RNA sequencing.

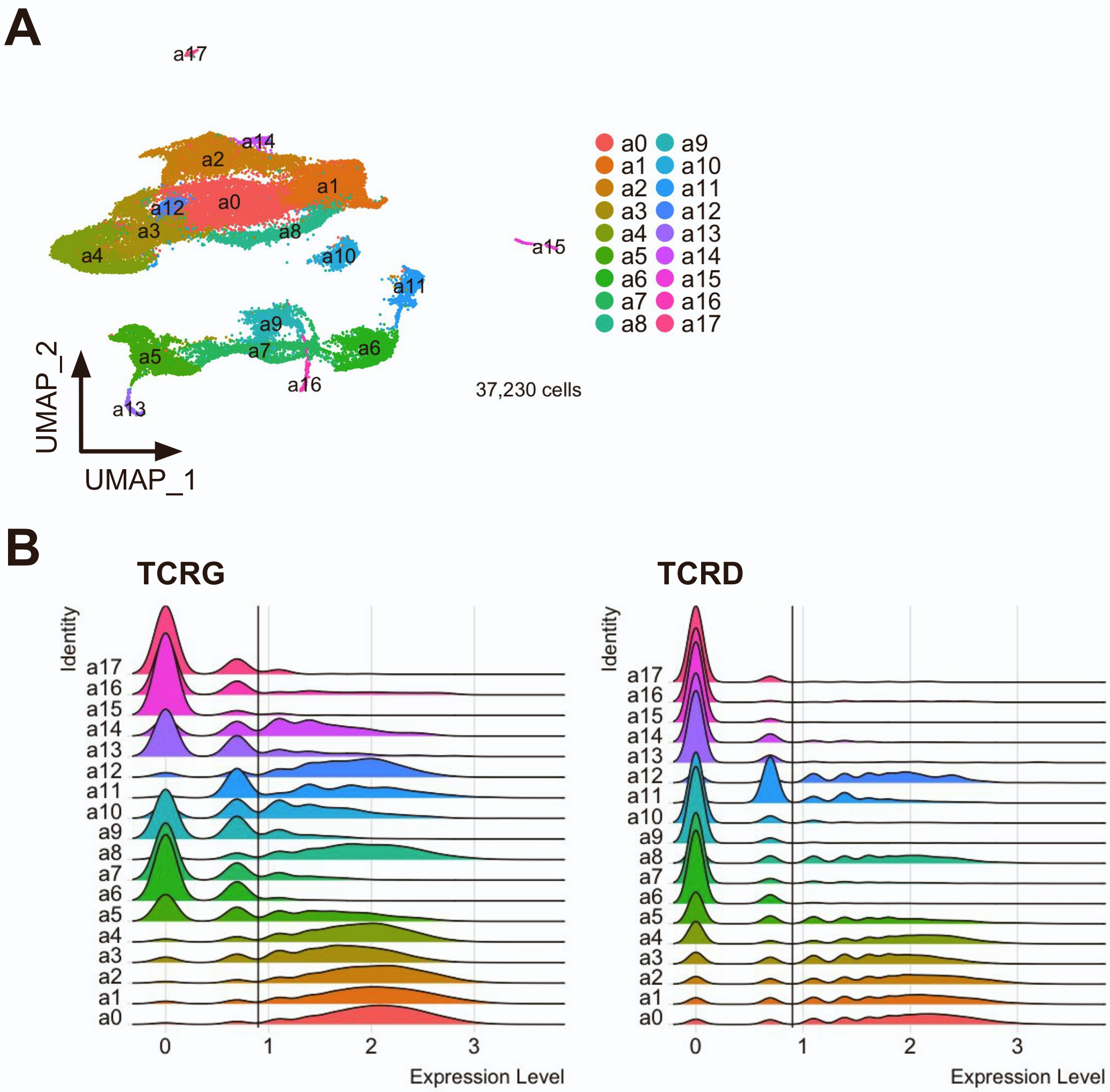

Figure S4. Identification of γδ T cells for downstream single-cell transcriptomic analysis.

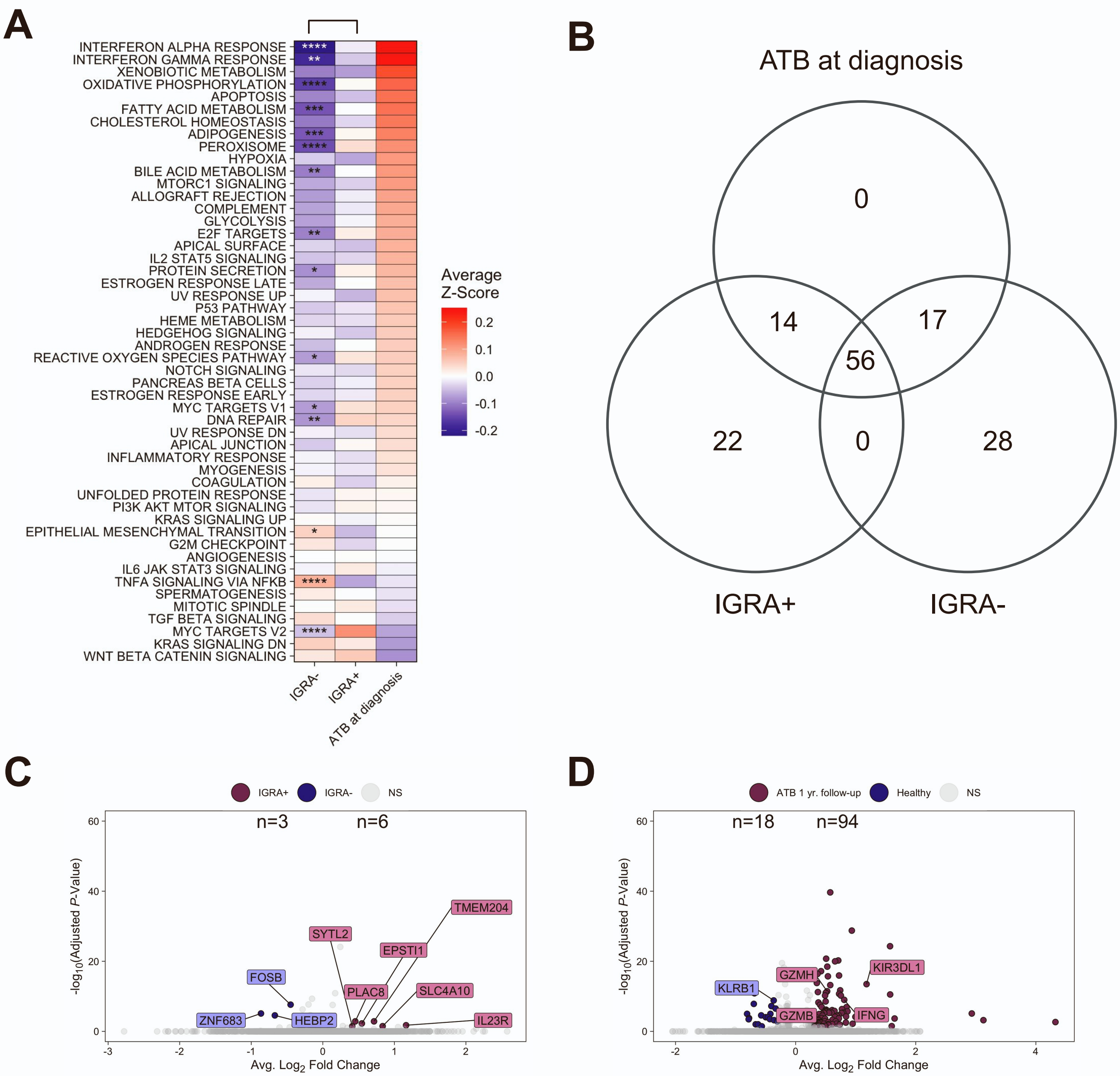

Figure S5. Additional pathway enrichment and differential expression analyses across clinical cohorts.

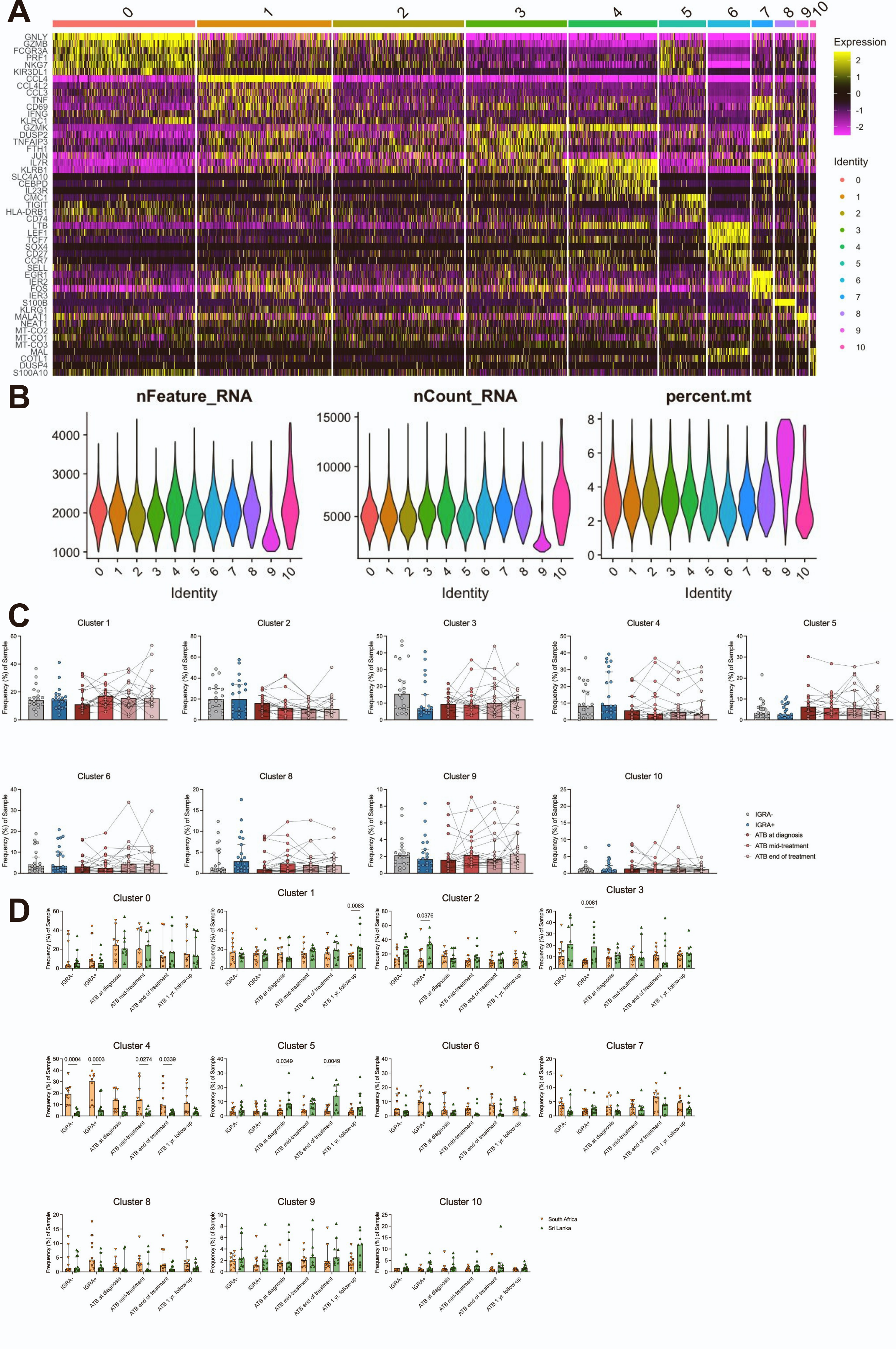

Figure S6. Cluster characterization, quality control, and sample distribution.

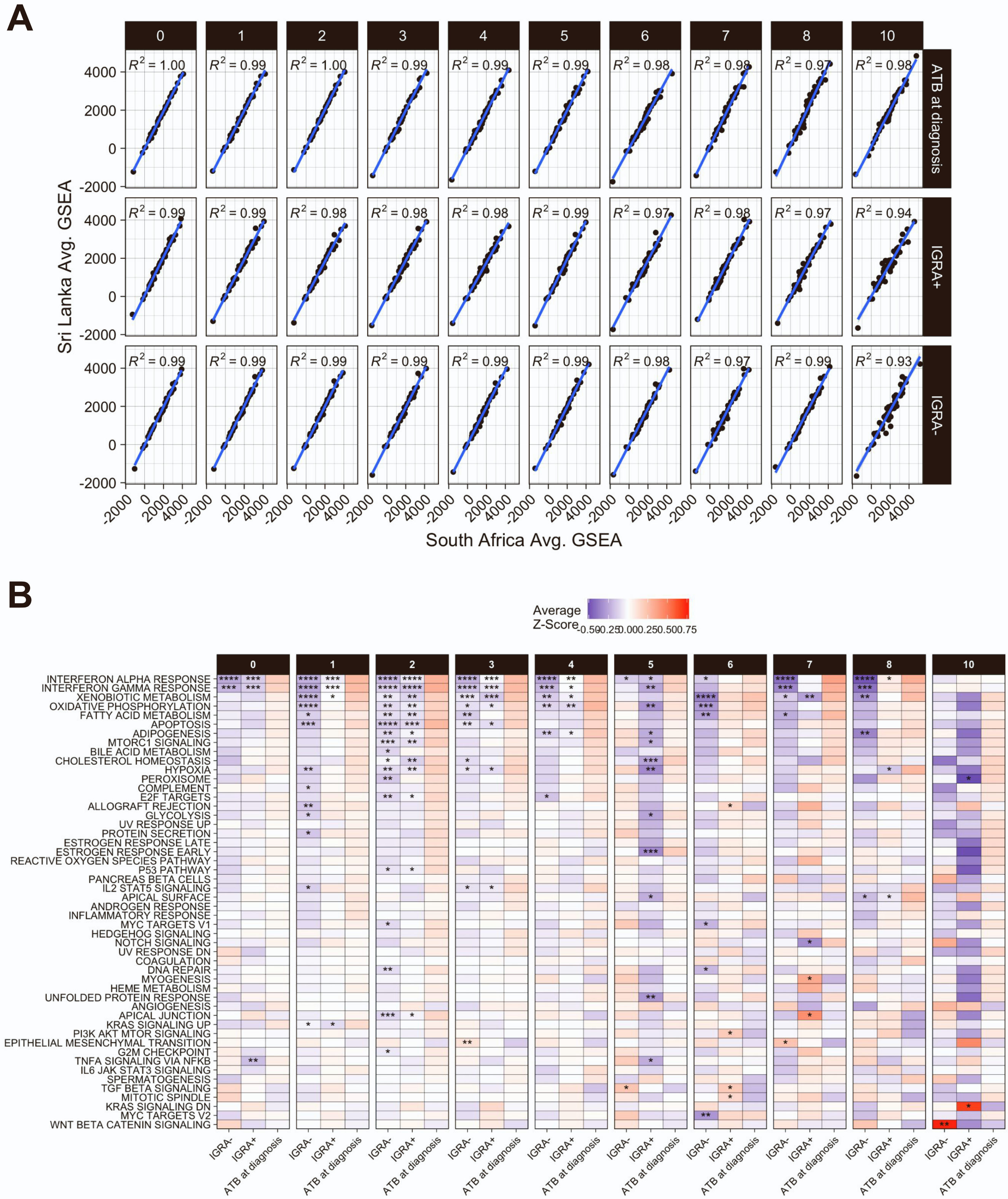

Figure S7. Additional analyses supporting pathway enrichment across geographical locations and cell clusters.

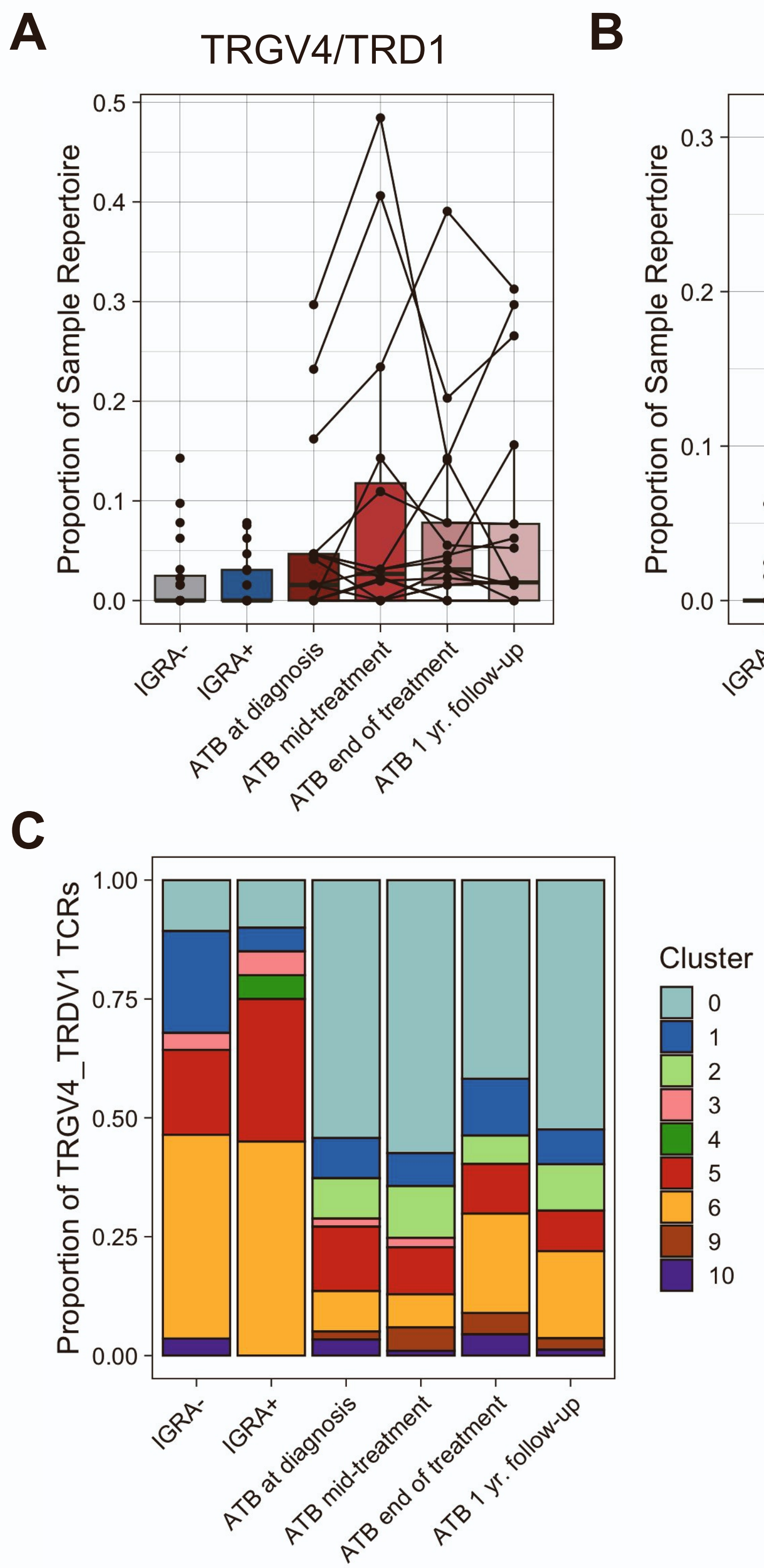

Figure S8. Distribution of *TRGV4*-associated TCR clonotypes across samples and cell clusters.

Donor	Cohort	Num_Cells
SUB415434	ATB at diagnosis	329
SUB415434	ATB mid-treatment	224
SUB415434	ATB end of treatment	106
SUB415434	ATB 1 yr. follow-up	279
SUB415435	ATB at diagnosis	1
SUB415435	ATB mid-treatment	101
SUB415435	ATB end of treatment	246
SUB415435	ATB 1 yr. follow-up	465
SUB415436	ATB at diagnosis	173
SUB415436	ATB mid-treatment	22
SUB415436	ATB end of treatment	40
SUB415436	ATB 1 yr. follow-up	71
SUB415437	ATB at diagnosis	72
SUB415437	ATB mid-treatment	181
SUB415437	ATB end of treatment	228
SUB415437	ATB 1 yr. follow-up	145
SUB415438	ATB at diagnosis	154
SUB415438	ATB mid-treatment	375
SUB415438	ATB end of treatment	384
SUB415438	ATB 1 yr. follow-up	50
SUB415440	ATB at diagnosis	226
SUB415440	ATB end of treatment	14
SUB415440	ATB 1 yr. follow-up	10
SUB415441	ATB at diagnosis	118
SUB415441	ATB mid-treatment	216
SUB415441	ATB end of treatment	249
SUB415441	ATB 1 yr. follow-up	167
SUB415442	ATB at diagnosis	108
SUB415442	ATB mid-treatment	162
SUB415442	ATB end of treatment	147
SUB415442	ATB 1 yr. follow-up	91
SUB415443	ATB at diagnosis	253
SUB415443	ATB mid-treatment	266
SUB415443	ATB end of treatment	39
SUB415443	ATB 1 yr. follow-up	178
SUB415444	IGRA-	390
SUB415445	IGRA-	43
SUB415446	IGRA+	53

SUB415447	IGRA+	146
SUB415448	IGRA-	320
SUB415449	IGRA-	117
SUB415450	IGRA-	236
SUB415451	IGRA-	77
SUB415452	IGRA+	313
SUB415453	IGRA-	86
SUB415454	IGRA-	76
SUB415455	IGRA+	16
SUB415456	IGRA+	33
SUB415457	IGRA-	47
SUB415458	IGRA-	2
SUB415459	IGRA+	215
SUB415460	IGRA+	69
SUB415461	IGRA+	1
SUB415462	IGRA+	59
SUB415463	IGRA+	71
SUB292861	ATB at diagnosis	116
SUB292861	ATB mid-treatment	93
SUB292861	ATB end of treatment	73
SUB292861	ATB 1 yr. follow-up	100
SUB292860	ATB at diagnosis	325
SUB292860	ATB mid-treatment	292
SUB292860	ATB end of treatment	241
SUB292860	ATB 1 yr. follow-up	312
SUB292858	IGRA+	312
SUB292857	IGRA+	96
SUB292856	IGRA-	293
SUB292855	IGRA+	303
SUB292854	IGRA+	199
SUB292852	IGRA+	120
SUB292851	IGRA+	182
SUB292850	IGRA+	90
SUB292849	IGRA+	160
SUB292848	IGRA+	206
SUB292847	ATB at diagnosis	254
SUB292847	ATB mid-treatment	325
SUB292847	ATB end of treatment	113
SUB292847	ATB 1 yr. follow-up	452

SUB292846	IGRA+	234
SUB292845	ATB at diagnosis	693
SUB292845	ATB mid-treatment	413
SUB292845	ATB end of treatment	321
SUB292845	ATB 1 yr. follow-up	93
SUB292840	ATB at diagnosis	46
SUB292840	ATB mid-treatment	190
SUB292840	ATB end of treatment	155
SUB292840	ATB 1 yr. follow-up	83
SUB292837	IGRA-	297
SUB292836	IGRA-	205
SUB292835	IGRA-	89
SUB292833	IGRA-	185
SUB292828	IGRA-	78
SUB292827	IGRA-	239
SUB292825	ATB at diagnosis	166
SUB292825	ATB mid-treatment	295
SUB292825	ATB end of treatment	271
SUB292825	ATB 1 yr. follow-up	247
SUB415464	ATB at diagnosis	404
SUB415464	ATB mid-treatment	385
SUB415464	ATB end of treatment	335
SUB415464	ATB 1 yr. follow-up	343
SUB415465	IGRA-	96
SUB402034	IGRA-	111
SUB402035	IGRA-	136
SUB292819	ATB at diagnosis	315
SUB292819	ATB mid-treatment	187
SUB292819	ATB end of treatment	50
SUB292819	ATB 1 yr. follow-up	228

Table S1. Number of $CD4^-CD8^-$ $\gamma\delta$ T cells per sample used in scRNAseq data analysis.

Comparisons	0	1	2	3	4	5	6	7	8	9	10
IGRA- vs. IGRA+	0.4723149	0.62631188	0.61670858	0.07419814	0.59689722	1	0.28257906	0.89920154	0.89529913	0.33322973	1
ATB_V1 vs. IGRA-	0.00044785	0.74775155	0.59870507	0.32019683	0.1386265	0.11089248	0.47334381	0.57250159	0.93398348	1	0.64852336
ATB_V2 vs. IGRA-	0.01815421	0.16749337	0.32606247	0.61910604	0.20919291	0.11650538	0.25919976	0.36229419	0.44123429	0.93906555	0.39079949
ATB_V3 vs. IGRA-	0.05162159	0.55872434	0.01933805	0.38834138	0.23330862	0.13174238	0.42777489	0.73361043	0.53989481	0.51818076	0.2826107
ATB_V4 vs. IGRA-	0.00567902	0.40912893	0.20868655	0.57279021	0.11370981	0.27283268	0.53847573	0.30396418	0.26153931	1	0.3826115
ATB_V1 vs. IGRA+	0.00072387	0.83783928	0.98833468	0.33463668	0.05919971	0.10401918	0.06640799	0.66172831	1	0.40104367	0.52338234
ATB_V2 vs. IGRA+	0.02923274	0.03860294	0.79558726	0.10350714	0.15595108	0.14748469	0.03357375	0.46153571	0.58897068	0.3008894	0.34032645
ATB_V3 vs. IGRA+	0.14707949	1	0.05928316	0.27283268	0.06714337	0.11667101	0.75600347	0.5586607	0.72791712	0.10232063	0.31280513
ATB_V4 vs. IGRA+	0.00324223	0.13334297	0.58852768	0.26374015	0.09268605	0.27241235	0.50787182	0.3826564	0.3826115	0.26224242	0.26349276

Table S5. Mann-Whitney U test results following centered log ratio normalization of sample frequencies per cohort and cluster.
